# Myths and Misconceptions of Airway Pressure Release Ventilation: Getting Past the Noise and on to the Signal

**DOI:** 10.3389/fphys.2022.928562

**Published:** 2022-07-25

**Authors:** Penny Andrews, Joseph Shiber, Maria Madden, Gary F. Nieman, Luigi Camporota, Nader M. Habashi

**Affiliations:** ^1^ R Adams Cowley Shock Trauma Center, University of Maryland School of Medicine, Baltimore, MD, United States; ^2^ University of Florida College of Medicine, Jacksonville, FL, United States; ^3^ Department of Surgery, SUNY Upstate Medical University, Syracuse, NY, United States; ^4^ Department of Adult Critical Care, Guy’s and St Thomas’ NHS Foundation Trust, Health Centre for Human and Applied Physiological Sciences, London, United Kingdom

**Keywords:** APRV, myth, airway pressure release ventilation (APRV), TCAV, time controlled adaptive ventilation

## Abstract

In the pursuit of science, competitive ideas and debate are necessary means to attain knowledge and expose our ignorance. To quote Murray Gell-Mann (1969 Nobel Prize laureate in Physics): “Scientific orthodoxy kills truth”. In mechanical ventilation, the goal is to provide the best approach to support patients with respiratory failure until the underlying disease resolves, while minimizing iatrogenic damage. This compromise characterizes the philosophy behind the concept of “lung protective” ventilation. Unfortunately, inadequacies of the current conceptual model–that focuses exclusively on a nominal value of low tidal volume and promotes shrinking of the “baby lung” - is reflected in the high mortality rate of patients with moderate and severe acute respiratory distress syndrome. These data call for exploration and investigation of competitive models evaluated thoroughly through a scientific process. Airway Pressure Release Ventilation (APRV) is one of the most studied yet controversial modes of mechanical ventilation that shows promise in experimental and clinical data. Over the last 3 decades APRV has evolved from a rescue strategy to a preemptive lung injury prevention approach with potential to stabilize the lung and restore alveolar homogeneity. However, several obstacles have so far impeded the evaluation of APRV’s clinical efficacy in large, randomized trials. For instance, there is no universally accepted standardized method of setting APRV and thus, it is not established whether its effects on clinical outcomes are due to the ventilator mode *per se* or the method applied. In addition, one distinctive issue that hinders proper scientific evaluation of APRV is the ubiquitous presence of myths and misconceptions repeatedly presented in the literature. In this review we discuss some of these misleading notions and present data to advance scientific discourse around the uses and misuses of APRV in the current literature.

## Introduction

“*Falsehood flies, and truth comes limping after it…….*”*—Jonathan Swift*. Similarly, a myth about airway pressure release ventilation (APRV) can be published, perpetuated, and believed as fact before science has a chance to get out of the laboratory. Some APRV myths stem from what intuitively seems reasonable when making a mental comparison between APRV and the current conceptual model of delivering “lung protective ventilation.” Unfortunately, this still revolves exclusively on the simplistic setting of a nominal and arbitrary value of “low” tidal volume (LV_T_) and levels of pressures which promote further shrinking of the “baby lung” (Marini and Gattinoni, 2020). Data increasingly show this model is not only incorrect but may contribute to the unacceptably high mortality rate of patients with moderate and severe acute respiratory distress syndrome (ARDS) (Amato et al., 2015; Costa et al., 2021; Goligher et al., 2021; Raschke et al., 2021). Additional myths and misconceptions are generated from the confused lumping of different ventilator modes and methods under an umbrella term of APRV and the differing ventilator behavior from various implementations by ventilator manufacturers.

**FIGURE 1:**
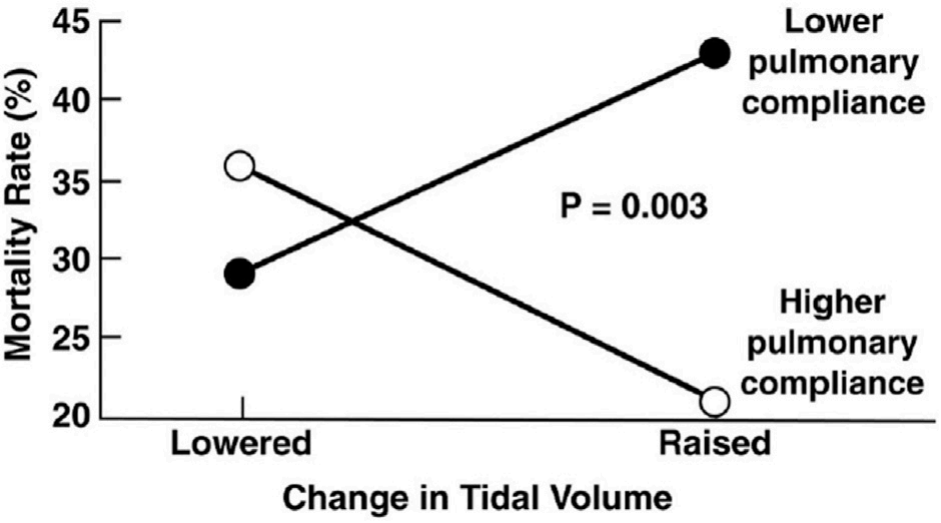
Data from the original ARMA Trial shows correlation between tidal volume (V_T_) and respiratory system compliance (C_RS_) of individual patients on mortality. Using the volume assist control mode a lower V_T_ reduced mortality with low C_RS_ where a higher V_T_ increased mortality in patients with high C_RS_. (Deans et al., 2005).

To scientifically study any ventilator mode, consistent methodology to set and adjust the mode is essential. This was clearly seen in the acute respiratory distress syndrome (ARDS) Network (ARDSNet) trial of low tidal volume ventilation study (ARMA) that used the volume assist–control (VAC) mode and compared lower with higher settings of tidal volumes (V_T_) and plateau pressures (Pplat) (ARDSNet 2000). Changing just these two parameters resulted in a significant reduction in mortality even when using the same mode. Of equal interest, in a subsequent analysis of 2,587 patients from the ARMA study that met criteria but were not enrolled for technical reasons, it was shown that high or LV_T_ will either increase or decrease mortality, depending on respiratory system compliance (C_RS_) of the individual patient as shown in Figure 1 (Deans et al., 2005). These initial data are further supported by more recent studies (Amato et al., 2015; Costa et al., 2021; Goligher et al., 2021; Raschke et al., 2021) and make it clear that a protective ventilation strategy can only be interpreted in the context of respiratory mechanics. Undoubtedly, even small changes in mode settings can have a significant impact on outcome depending on the degree of lung pathophysiology and patient heterogeneity suggesting a need for personalization of lung protective strategies (Nieman et al., 2017a; Pelosi et al., 2021; Cheng et al., 2022).

**FIGURE 2:**
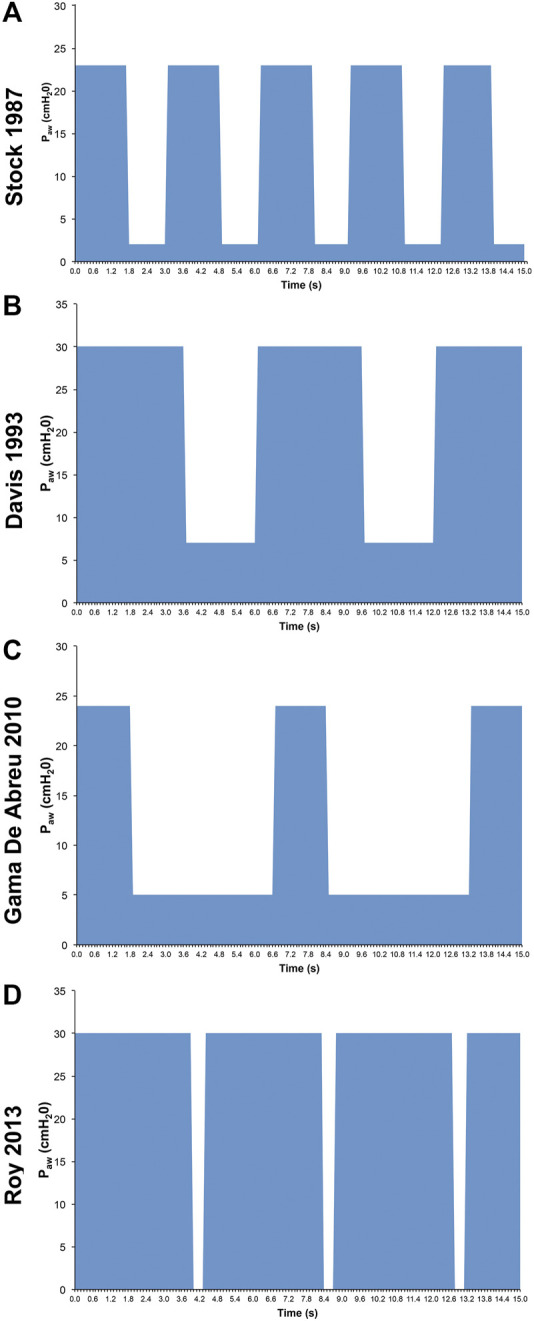
Airway Pressure Release Ventilation (APRV) Pressure/Time waveforms from 4 studies: **(A)** Stock et al., 1987 set time at P_Low_ (T_Low_) of 1.27s (Stock et al., 1987); **(B)** Davis et al., 1993 used an increased inspiratory to expiratory ratio (Davis et al., 1993); **(C)** Gama de Abreau 2010 simulated conventional ventilation (Gama de Abreau 2010); **(D)** Roy et al., 2013a used the Time Controlled Adaptive Ventilation method (Roy SK. et al., 2013). This illustrates the wide variability in methods used to set APRV, which may dramatically impact outcome (Jain et al., 2016).

**FIGURE 3:**
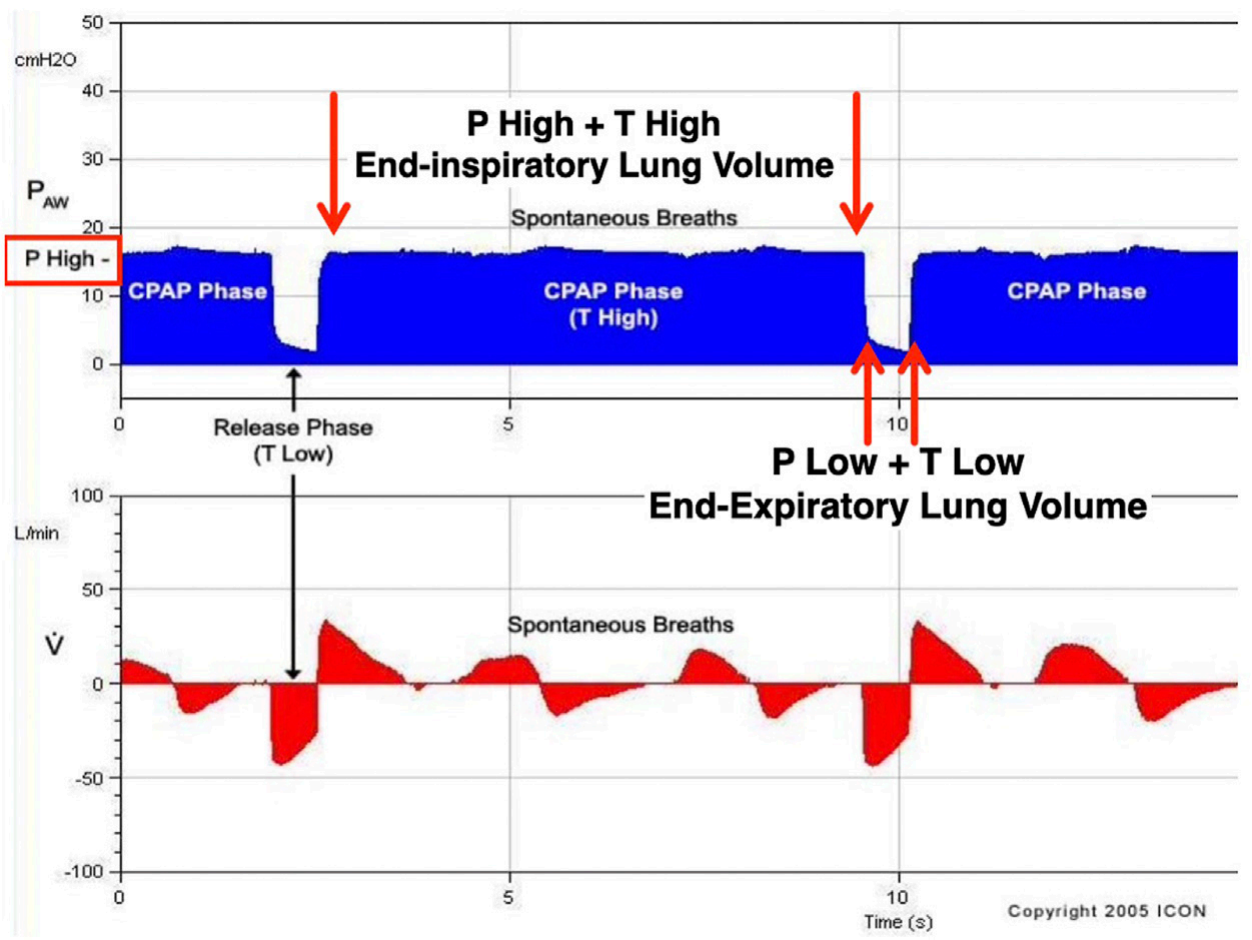
Airway Pressure Release Ventilation (APRV) is a pressure-limited, time-cycled mode. The Time Controlled Adaptive Ventilation (TCAV^TM^) method of setting the APRV mode includes the following settings: 1) upper airway pressure (P_High_); 2) lower airway pressure (P_Low_); 3) time spent at P_High_ (T_High_); and 4) time spent at P_Low_ (T_Low_). Combined, P_High_ and T_High_ form the continuous positive airway pressure (CPAP) Phase and impact end-inspiratory lung volume. The CPAP Phase releases to the combined P_Low_ and T_Low_, which form the Release Phase and impact end-expiratory lung volume. During the TCAV^TM^ method of APRV, the ventilator cycles between the CPAP and Release Phases. During the release phase, the T_Low_ set to terminate at 75% of the peak expiratory flow rate halts alveolar instability. Subsequently, the CPAP Phase maintains alveolar stability and recruits lung volume over time (hours to days).

Although APRV has been available on commercial ventilators since 1987, the method of applying the mode has varied widely in medical literature and clinical practice (Figure 2) (Jain et al., 2016; Habashi et al., 2021). Currently, APRV is an ill-defined initialism which identifies a mode without a consistent method of application. In fact, APRV is often used as a synonym for the biphasic positive airway pressure (BIPAP) mode so much so as to be found in the literature often indicated as a meaningless BIPAP/APRV mode (Neumann et al., 2002; Dries and Marini, 2009; Kallet 2011; Daoud et al., 2012). Subsequently, the outcome in both basic science and clinical studies using APRV with different settings (Jain et al., 2016) has led to further confusion on the relative efficacy of individual components of the APRV settings–particularly the value of inspiratory (T_High_) and expiratory (T_Low_) time settings (Habashi et al., 2022). APRV was originally described as continuous positive airway pressure (CPAP) with a release phase. There are four basic settings to control in APRV other than FiO_2_: 1) P_High_ (inspiratory pressure similar to Pplat; 2) T_High_ (duration of inspiratory time) - when combined with the P_High_ controls end-inspiratory lung volume and referred to as the CPAP Phase; 3) P_Low_ (expiratory pressure similar to PEEP); 4) T_Low_ (duration of expiratory time) - when combined with the P_Low_ controls end-expiratory lung volume (EELV) and referred to as the Release Phase (Figure 3). The method to set and adjust APRV that has been used most clinically, spanning over 30 years, and best studied consistently in translational animal models that exceed American Thoracic Society animal model guidelines (Matute-Bello et al., 2011) is the Time Controlled Adaptive Ventilation (TCAV™) method (Roy et al., 2012; Roy S. et al., 2013; Roy SK. et al., 2013; Andrews et al., 2013a; Andrews et al., 2013b; Emr et al., 2013; Kollisch-Singule et al., 2014a; Kollisch-Singule et al., 2014b; Kollisch-Singule et al., 2015; Kollisch-Singule et al., 2016a; Kollisch-Singule et al., 2019; Smith et al., 2015; Jain et al., 2017; Silva et al., 2018; Mahajan et al., 2019; Al-khalisy et al., 2020; Bates et al., 2020; de Magalhã;es et al., 2021; Vasconcellos de Oliveira et al., 2022).

The TCAV™ method emphasizes *time control* of the upper and lower pressures and an adaptive methodology to personalize a lung protective strategy for each patient’s respiratory mechanics throughout the evolution—or resolution—of their lung disease process (Habashi 2005; Habashi et al., 2011; Habashi and Andrews, 2013; Habashi et al., 2019). Unique to the TCAV™ method of setting APRV is using passive exhalation without a set PEEP and analyzing the slope of the expiratory flow-time curve (SLOPE_EF_) (Dixon and Brodie, 1903; Rahn et al., 1946; Mead and Whittenberger, 1953; Brody, 1954; Comroe 1954; Brody and DuBois, 1956; McIlroy et al., 1963; Bergman 1966; Grimby et al., 1968; Ashutosh and Keighley, 1978; Behrakis et al., 1983; Richardson et al., 1989; Baydur and Carlson, 1994; Brunner et al., 1995; Guttmann et al., 1995; Nassar et al., 2012) to personalize V_T_ to C_RS_, which has been validated experimentally and clinically (Roy et al., 2012; Roy S. et al., 2013; Roy SK. et al., 2013; Andrews et al., 2013b; Emr et al., 2013; Kollisch-Singule et al., 2014a; Kollisch-Singule et al., 2014b; Kollisch-Singule et al., 2015; Kollisch-Singule et al., 2016a; Kollisch-Singule et al., 2016b; Kollisch-Singule et al., 2019; Kollisch-Singule et al., 2020; Smith et al., 2015; Jain et al., 2017; Silva et al., 2018; Mahajan et al., 2019; Al-khalisy et al., 2020; Bates et al., 2020; de Magalhã;es et al., 2021; Vasconcellos de Oliveira et al., 2022). The T_Low_ is tuned to the elastic recoil of the respiratory system (E_RS_) to halt alveolar collapse aiding in distal airspace stability and when coupled with the P_High_ and T_High_, the CPAP phase gradually normalizes lung volume over hours to days (Kollisch-Singule et al., 2014a; Boehme et al., 2015; Kollisch-Singule et al., 2016a). This allows the lungs of each patient to determine the time-course to normalize lung volume rather that the clinician forcing it open such as with recruitment maneuvers (RMs).

We reviewed the current relevant literature identified using OvidSP and the National Library of Medicine’s MEDLINE database via PubMed to locate published papers using APRV and identified myths and misconceptions consistently seen in the literature. This review discusses 10 myths and misconceptions about APRV, which are largely based on opinions or methodologic inconsistencies and lack evidence to support those inaccurate claims. Additionally, we found that many APRV myths originate in review articles, editorials, or the discussion section of papers. In other words, they reflect inferences, extrapolations, personal beliefs including hyperbole yet lack the furtherance of credible scientific evidence. These opinions then become an echo chamber that reverberates in the literature and become self-evident truths.

## Myth #1—Airway Pressure Release Ventilation is too Difficult to use

Several papers include statements such as: “APRV evolved into a highly sophisticated, physiology-driven, dynamic mechanical breath profile with precise settings, which might cause a possibility of knowledge bias by the staff” (Zhong et al., 2020) and “APRV is more complex than it appears to be. It requires a lot more knowledge and skill than may be apparent from the descriptions in the literature (Chatburn et al., 2016).” These and other statements (MacIntryre, 2011) lead the reader to believe APRV is too difficult to use for the average practicing clinician. Further, it has been suggested that a simulator is the only practical way to gain understanding of APRV because equivalent experience with real patients could take years and put a lot of people at risk (*sic*) (Chatburn et al., 2016). This insinuates there is no risk in using any other ventilator mode nor is skill required and is dismissive of the mortality rate with current approaches to manage ARDS that continue to range from 35 to 49% (Villar et al., 2014; Bellani et al., 2016; Cavalcanti et al., 2017). Further, mechanical ventilation training in general suffers from a lack of structure, is non-standardized -leading to poor training and knowledge of mechanical ventilation- and often leaves the trainee dissatisfied (Goligher et al., 2012; Wilcox et al., 2016; Keller et al., 2019; Seam et al., 2021). Add to this the existence of a learning curve for any new medical device, procedure, technique, or ventilator mode including APRV (Govindarajulu et al., 2017). Indeed, like any other mode, using APRV for the first time without a general understanding of the rationale and settings on a critically ill and unstable patient with severe ARDS who is failing ‘conventional therapies’ may not be as successful as when applied by providers who have experience and use it daily as their primary mechanical ventilation strategy. In actuality, APRV has already been used successfully on tens of thousands of patients for over 30 years and continues to be a part of daily care in many hospitals amassing a large amount of empirical data (Sadowitz et al., 2011; Andrews et al., 2013b; Mallory and Cheifetz, 2020; Rola and Daxon, 2022). It is understandable that users who have never actually used APRV or are unfamiliar with this way of thinking about mechanical ventilation may consider it too difficult (Nieman et al., 2017a; Nieman et al., 2017b; Nieman et al., 2018b; Nieman et al., 2020a; Nieman et al., 2020b). However, there are many things in medicine and clinical practice that seem far more difficult but are used with proper education and training such as high frequency oscillatory ventilation (HFOV) and extracorporeal membrane oxygenation. In fact, after the ARMA trial, clinicians at the original 10 ARDSNet sites were surveyed on their use and experience of the ARDSNet protocol (Rubenfeld et al., 2004). The survey showed experienced bedside clinicians perceived important barriers to implementing lung protective ventilation. Obviously, such limitations can be overcome with education, training and experience and is not seen exclusively with APRV.

Although over emphasized, concern for APRV settings permeates the literature yet the more conventional approach to ventilator settings such as V_T_, respiratory rate (RR), and PEEP remains controversial despite decades of research and debate (Deans et al., 2005; Amato et al., 2015; Sahetya et al., 2017; Algera et al., 2018; Costa et al., 2021; Goligher et al., 2021; Pelosi et al., 2021; Abrams et al., 2022; Dianti et al., 2022). In addition, important elements of mechanical ventilation such as RR, inspiratory time and flows, and expiratory time and flows are generally not reported or ignored–whereas they are essential components of the total energy delivered to the lung (Gattinoni et al., 2016; Bates et al., 2020) and the combination of these factors can promote lung healing or injury.

As for being a highly sophisticated ventilation mode or too difficult to learn, APRV does not require an in depth understanding of distinctive settings such as frequency (cycles per second) set in Hz and amplitude/power nor the use of a dedicated ventilator such as with HFOV. In fact, APRV is available on almost all intensive care unit (ICU) ventilators as either a standard mode or an option. Like any ventilator mode, APRV uses the same elements: 1) pressure, 2) flow and 3) volume. The key is the personalized configuration of the elements to create a stable airway pressure profile (CPAP Phase) that offers a rate (Release Phase). The airway profile of APRV highlights and leverages the use of time in a time-dependent viscoelastic organ such as the lung (Nieman et al., 2017a; Nieman et al., 2017b; Nieman et al., 2020a; Nieman et al., 2020b). Standard APRV settings include: 1) upper airway pressure (P_High_), 2) time spent at P_High_ (T_High_) [combined these define the CPAP Phase]; 3) lower airway pressure (P_Low_), and 4) time spent at P_Low_ (T_Low_) [combined these define the Release Phase] (Figure 3). With the TCAV™ method, the P_High_ is set to Pplat as you would in a pressure mode. The P_Low_ [typically referred to as PEEP in other ventilatory modes] is set to 0 cmH_2_O because EELV is directly controlled with time instead of a set PEEP. This simplifies the quest for the optimal PEEP which has remained elusive despite over 50 years of study and debate and still lacks a refined approach to personalization (Sahetya et al., 2017). The adjustment for time is also simplified as the T_Low_ is used to balance the E_RS_ by retaining EELV and preventing expiratory collapse (Kollisch-Singule et al., 2014a). Setting and personalizing the T_Low_ to achieve termination of the expiratory flow (*E*_FT_) at 75% of the peak expiratory flow (*E*_PF_) rate in normal to high E_RS_ -and 25% with low E_RS_ such as chronic obstructive pulmonary disease (COPD)—captures the majority of the closing time constants, thereby maintaining alveolar stability and ductal patency (Kollisch-Singule et al., 2014a, b; Vasconcellos de Oliveira et al., 2022). This personalization of the T_Low_ simplifies pairing V_T_ to C_RS_, and provides a real-time, bedside, non-invasive assessment using the SLOPE_EF_, which are all congruent with evolving or resolving changes in respiratory system mechanics. Since V_T_ does not correlate well with predicted body weight (PBW) in ARDS patients and appears that normalization of V_T_ to C_RS_ (i.e., driving pressure) relates to better outcome (Amato et al., 2015; Costa et al., 2021; Goligher et al., 2021; Pelosi et al., 2021), T_Low_ personalization of V_T_ to C_RS_ may be easier for real-time bedside monitoring and prove beneficial (Nieman et al., 2017a; Nieman et al., 2017b; Nieman et al., 2020a; Nieman et al., 2020b; Pelosi et al., 2021; Cheng et al., 2022; Habashi et al., 2022). Once the recoil forces of the lung are neutralized with the T_Low_, the T_High_ is left to adjust for ventilation by controlling RR, which is common to all ventilator modes.

## Myth #2—Airway Pressure Release Ventilation Causes Barotrauma

One of the most common myths regarding APRV is that it causes barotrauma (Myers and Macintyre, 2007; Dries and Marini, 2009; Esan et al., 2010; Kallet 2011; Daoud et al., 2012; Mireles-Cabodevila and Kcmarek, 2016; Hirshberg et al., 2018; Kami et al., 2019), yet is not supported by scientific literature. We are not saying barotrauma does not occur with APRV, but we are saying it does not happen more frequently than in any other ventilatory condition–including in patients receiving non-invasive ventilation or high flow nasal cannula (Hamouri et al., 2021; Palumbo et al., 2021; Shrestha et al., 2022). In fact, there is no evidence demonstrating any component (alone or in combination) is the sole cause of barotrauma.

It would be difficult to establish causality solely from a specific ventilator setting or mode as barotrauma is multifactorial including the population heterogeneity, severity, and inhomogeneity of lung disease on which settings are applied. In fact, a study of 5,183 patients showed no correlation between barotrauma and the mode of ventilation or ventilator settings (Anzueto et al., 2004). Further, in 30 years of large randomized controlled trials (RCTs) comparing various ventilator modes, settings and parameters including 6 ml/kg vs 12 ml/kg V_T_ (ARDSNet, 2000), low vs high PEEP (Brower et al., 2004), and low vs high mean airway pressure (Paw) (Ferguson et al., 2013; Young et al., 2013) there has been no direct relationship linking barotrauma with a specific ventilator mode or settings. Additionally, a systematic review and meta-analysis of eight RCTs comparing higher versus lower PEEP strategies enrolling 2,728 patients with ARDS showed no difference in barotrauma rates (Fan et al., 2017). One exception is the 2017 Alveolar Recruitment for Acute Respiratory Distress Syndrome Trial where a significant difference in barotrauma rates were seen between the group receiving lung RM with PEEP titration up to 45 cmH_2_O (5.6%) compared to the low PEEP group (1.6%) (Cavalcanti et al., 2017).

The potential for barotrauma seems primarily associated with the severity of underlying (acute or chronic) lung disease, which may be aggravated by mechanical ventilation (Anzueto et al., 2004). More recently, barotrauma rates have been reported to occur with greater frequency in COVID related ARDS (CARDS) but not specific to any one ventilator mode (McGuinness et al., 2020; Gazivoda et al., 2021; Hamouri et al., 2021; Rajdev et al., 2021; Udi et al., 2021; Belletti et al., 2022; Shrestha et al., 2022). In a systematic review and meta-analysis, a linear association of increased barotrauma incidence with increasing disease severity was observed in COVID-19 patients requiring various forms of invasive and non-invasive respiratory support (Shrestha et al., 2022). Despite this increased risk of barotrauma with COVID-19, no difference in barotrauma was seen between APRV or ARDSNet low V_T_ (LV_T_) in recent study of CARDS patients (Ibarra-Estrada et al., 2022).

To date, in RCTs comparing APRV with other ventilator modes where pneumothorax or pneumomediastinum was reported, there was no increased rate of barotrauma (Maxwell et al., 2010; Lim et al., 2016; Ganesan et al., 2018; Hirshberg et al., 2018; Lim and Litton, 2019; Zhong et al., 2020; Ibarra-Estrada et al., 2022). Conversely, Maxwell et al. (2010) showed the rate of pneumothorax was lower with APRV (0%) when compared with LV_T_ (3.1%) and a meta-analysis of seven RCTs with 405 eligible patients presented no statistical difference between LV_T_ and APRV in the incidence of pneumothorax (Zhong et al., 2020). In addition, a systematic review suggests mortality appears to be lower with APRV and no evidence of increased risk of barotrauma or other adverse consequences with APRV compared to LV_T_ in ARDS patients (Lim and Litton, 2019). Lastly, in three clinically applicable porcine models of sepsis-induced ARDS (Roy et al., 2012; Roy S. et al., 2013; Roy SK. et al., 2013; Kollisch-Singule et al., 2015) and a porcine neonatal infant respiratory distress syndrome model (Kollisch-Singule et al., 2016a) no barotrauma was noted, and lung injury prevented when using APRV.

**FIGURE 4:**
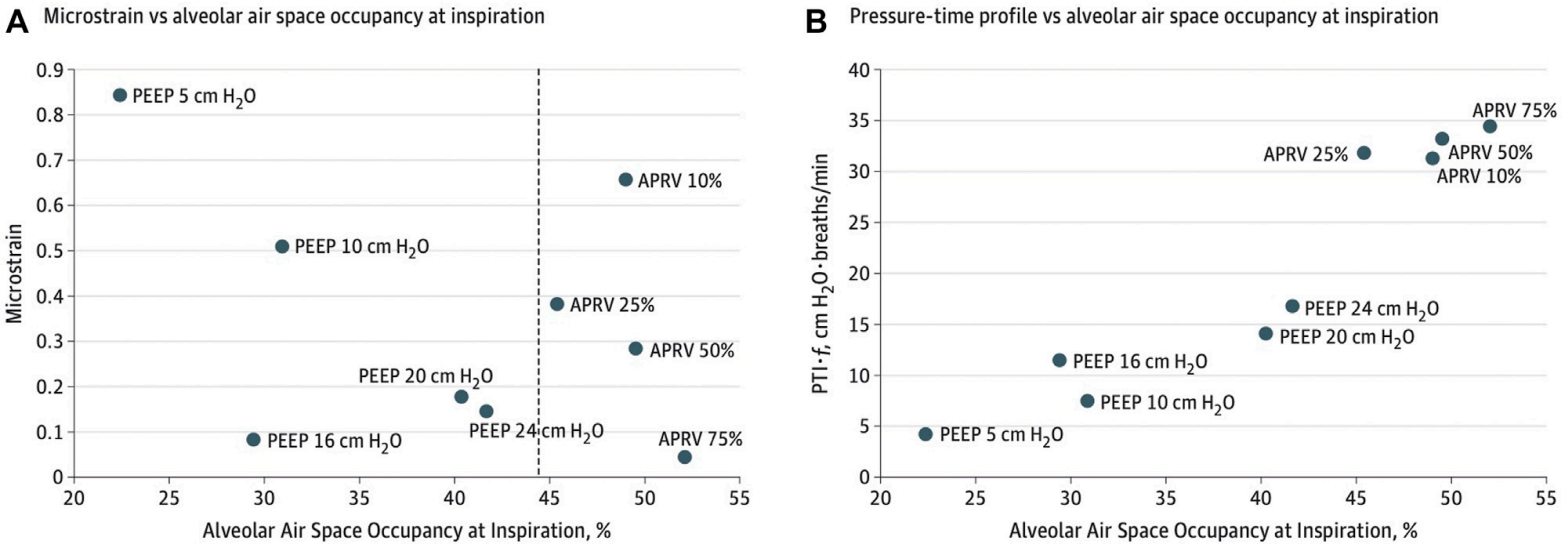
**(A)** Microstrain vs alveolar air space occupancy (Aa) at inspiration. The dashed line shows the difference in Aa between airway pressure release ventilation (APRV) and controlled mandatory ventilation. **(B)** Normalized pressure-time profile over a minute vs Aa at inspiration. PEEP indicates positive end-expiratory pressure and % for APRV indicate ratio of termination of peak expiratory flow rate to peak expiratory flow rate.

**FIGURE 5:**
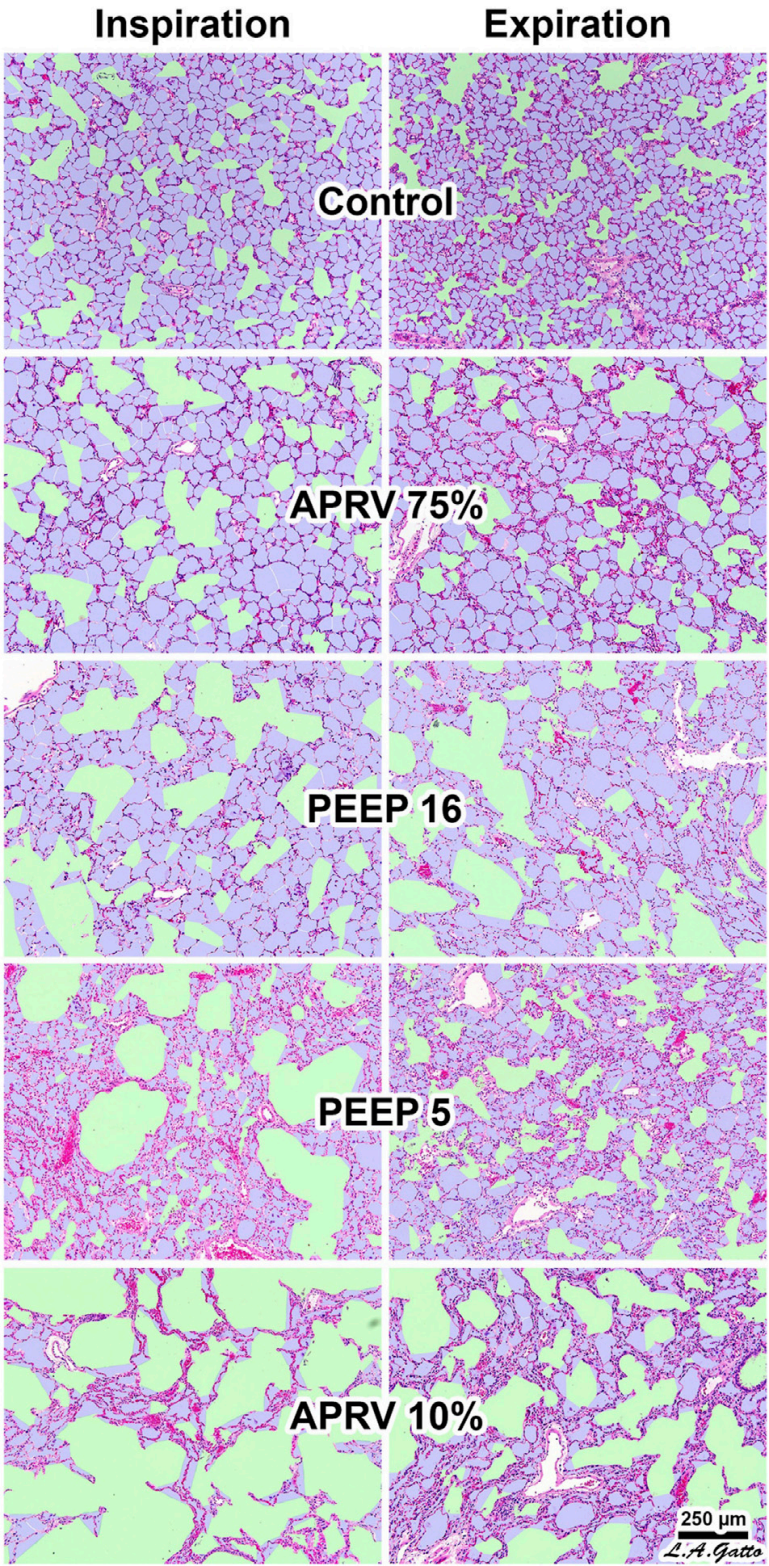
The Airway Pressure Release Ventilation (APRV) 75% group produced the greatest alveolar air space occupancy (Aa) at both inspiration and expiration (I/E), with values similar to control (*p* > 0.05) and resulted in the least conducting airway micro-strain. The conducting airway air space occupancy (Ca) to alveolar air space occupancy Aa, Ca/Aa at I/E, closely matched uninjured normal lung terminal airway gas distribution. The APRV 10% (T_Low_ extended) group had the least Aa at both I/E and the greatest conducting airway micro-strain suggesting precise control of time is critical. In the conventional mechanical ventilation group increasing PEEP from 5 to 16 cmH_2_O resulted in a greater degree of Ca rather than increasing Aa at I/E, suggesting increasing levels of PEEP primarily distend conducting airways rather than recruit alveolar gas and unable to restore the normal lung Ca/Aa.

**FIGURE 6:**
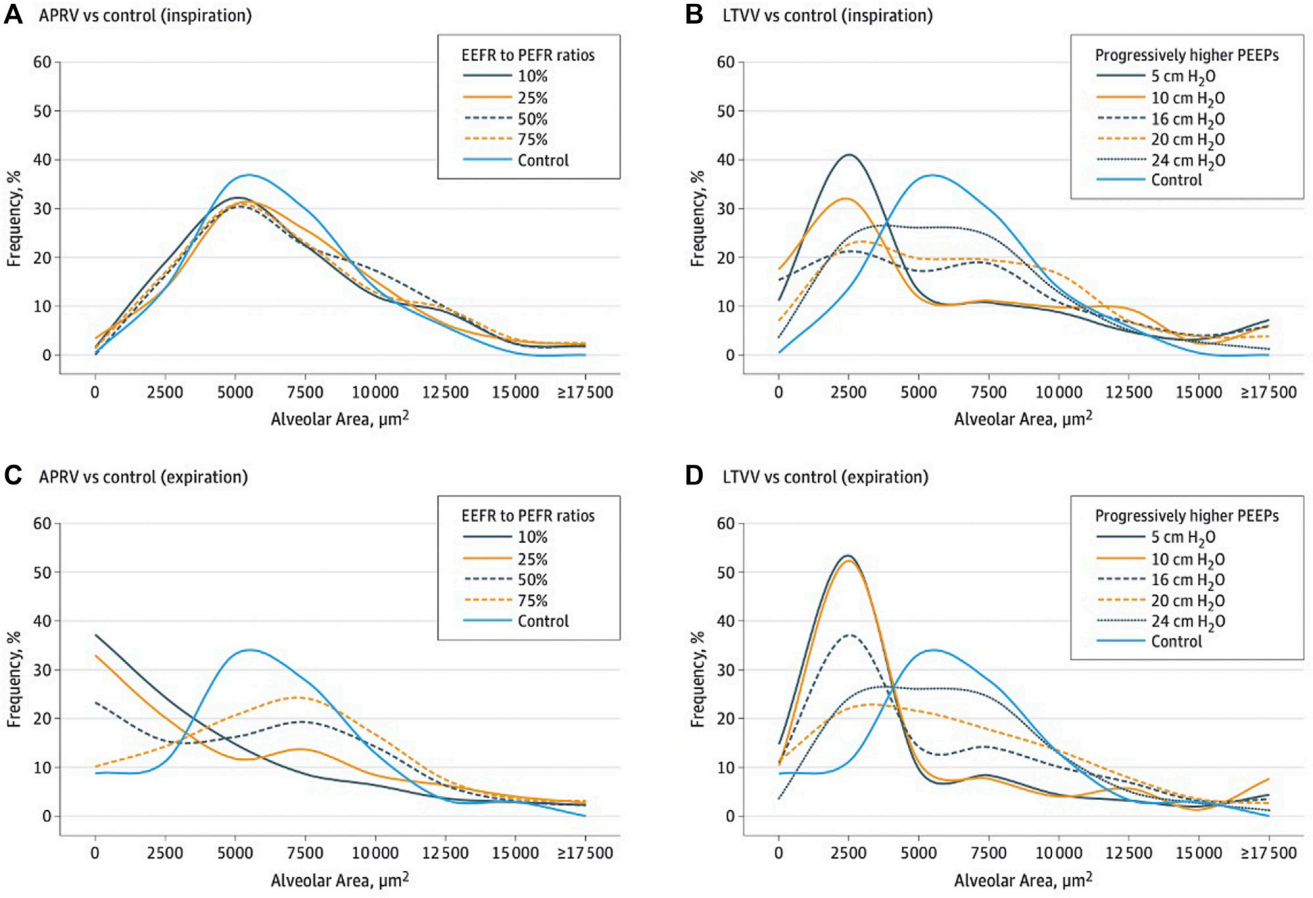
Histogram overlying normal and injury alveolar area and frequency of distribution reflecting alveolar heterogeneity post lung injury. **(A,B)** show inspiration histogram with normal pre-injury (blue line) where remainder lines are post-injury demonstrating APRV normalizes post-injury heterogeneity. The LV_T_ group showing V_T_ with various positive end-expiratory pressure (PEEP) levels (5 to 24 cmH_2_O) was not able to restore pre-injury homogeneity. **(C,D)** show expiration histogram with normal pre-injury (blue line) where remainder are post-injury demonstrating APRV normalizes post-injury heterogeneity. The LV_T_ group with various PEEP levels was not able to restore pre-injury.

Experimentally, micro-strain studies using APRV with the TCAV™ method vs LV_T_ suggest APRV has the lowest strain on distal air spaces (Figure 4) (alveoli and ducts), minimizes ductal dilatation (Figure 5) and restores alveolar homogeneity (Figure 6) after heterogenous lung injury when compared to LV_T_ with PEEP up to 24 cmH_2_O (Kollische-Singule et al., 2014a; Kollische-Singule et al., 2014b; Kollische-Singule et al., 2015). These studies suggest lung tissue strain is lower with the TCAV™ method and could be favorable to lower barotrauma rates. In summary, the underlying lung disease is the key risk for barotrauma (Anzueto et al., 2004; McGuinness et al., 2020; Gazivoda et al., 2021; Hamouri et al., 2021; Rajdev et al., 2021; Udi et al., 2021; Shrestha et al., 2022) and is therefore difficult to implicate any one ventilator mode or setting.

## Myth #3—Airway Pressure Release Ventilation Generates High Tidal Volumes Leading to Volutrauma

Several opinion papers (Kallet 2011; Modrykamien et al., 2011; Daoud et al., 2012) reference studies implying that APRV itself generates high V_T_ (Räsänen et al., 1991; Neumann et al., 2002; Varpula et al., 2004), which could potentially contribute to volutrauma. However, these studies demonstrate settings chosen by the operator (and not the mode) generated the high V_T_ and yet reported no evidence of volutrauma. For instance, in the Räsänen et al. (1991) study, it was not mentioned that although the V_T_ in the APRV group was 9 ml/kg, it was significantly lower than the conventional positive pressure ventilation group, which was 12 ml/kg. In the 2004 study by Varpula et al. (2004), APRV is singled out for high V_T,_ but a key point not mentioned is that both groups (synchronized intermittent mandatory ventilation-pressure control/pressure support (SIMV-PC/PS) and APRV) targeted V_T_ 8–10 ml/kg with no difference in V_T_ between modes. Interestingly, although these opinion papers reference Varpula et al. (2004) for high V_T_ in APRV, they neglect to cite a 2003 study [also by Varpula] comparing the same modes but the V_T_ in the APRV group was significantly lower than SIMV-PC/PS (Varpula et al., 2003). Some authors (Modrykamien et al., 2011) suggest an unvalidated claim of setting T_Low_ (*sic*) “40% of *E*_FP_ (around 0.6–0.8 s).” A T_Low_ of 40% of *E*_FP_ would not only assure a larger V_T_ than TCAV™ 75% but increases distal air space atelectrauma and induces lung injury (Kollische-Singule et al., 2014a; Kollische-Singule et al., 2014b; Kollische-Singule et al., 2016a; Jain et al., 2017). Lastly, a study by Neumann et al. (2002) is also frequently referenced regarding V_T_ greater than 1 L and large pleural pressure swings leading to large transpulmonary pressures that could contribute to volutrauma and ventilator induced lung injury (VILI) (Esan et al., 2010; Maxwell et al., 2010; Kallet 2011; Modrykamien et al., 2011; Daoud et al., 2012). However, what is not discussed is release times (T_Low_) of up to 2.5 s were used, creating large V_T_ unlike when they decreased T_Low_ to 0.5 s (typically used with the TCAV™ method of APRV) and the subsequent decrease in V_T_ when T_Low_ was decreased from 2.5 to 0.5 s.

If the operator targets a V_T_, then the mode cannot be blamed if this V_T_ is realized. Like any ventilator mode, high V_T_ may be generated with APRV as a result of variable methodologies as seen in several APRV studies (Jain et al., 2016). However, unlike the 2000 ARDSNet trial there have been no APRV studies linking an increase in mortality between groups even when V_T_ exceeds 6 ml/kg (Maxwell et al., 2010; Lim et al., 2016; Ganesan et al., 2018; Hirshberg et al., 2018; Lim and Litton, 2019; Zhong et al., 2020; Ibarra-Estrada et al., 2022).

With any pressure format mode of mechanical ventilation, the user selects the applied pressure and subsequent V_T_ is dependent on factors such as C_RS_, gas volume, airway resistance (R_AW_), and structural homogeneity of the lung. Therefore, the healthier the lung with a near normal C_RS_, the more likely the V_T_ will increase beyond the “magic” number of 6 ml/kg. For instance, if V_T_ in VAC is set to 12 ml/kg, then high V_T_ will be generated and if set to 6 ml/kg, then LV_T_ will be generated. The fact that V_T_ and settings are determined more by mechanics than by guidelines is evident in the recent re-analysis of the LUNG-SAFE data where patients with a greater C_RS_ received higher V_T_ (averaging 8.5 ml/kg PBW) compared to patients with low C_RS_ who received lower V_T_ (averaging 7.5 ml/kg PBW) (Goligher et al., 2021). Which patients were ventilated more protectively? The value of driving pressures (∆P) reveal that patients apparently ventilated more protectively (based on lower recorded values of V_T_) were in fact exposed to significantly higher ∆P and therefore at higher risk given that ∆P—not V_T_—is associated with greater risk of death (Amato et al., 2015; Bellani et al., 2016; Goligher et al., 2021). In addition, assigning very low V_T_ to patients with normal C_RS_ and R_AW_ leads to more asynchronies, breath stacking and ultimately higher risk of death (Deans et al., 2005; Bellani et al., 2016; Cavalcanti et al., 2017; Costa et al., 2021; Goligher et al., 2021; Raschke, et al., 2021).

**FIGURE 7:**
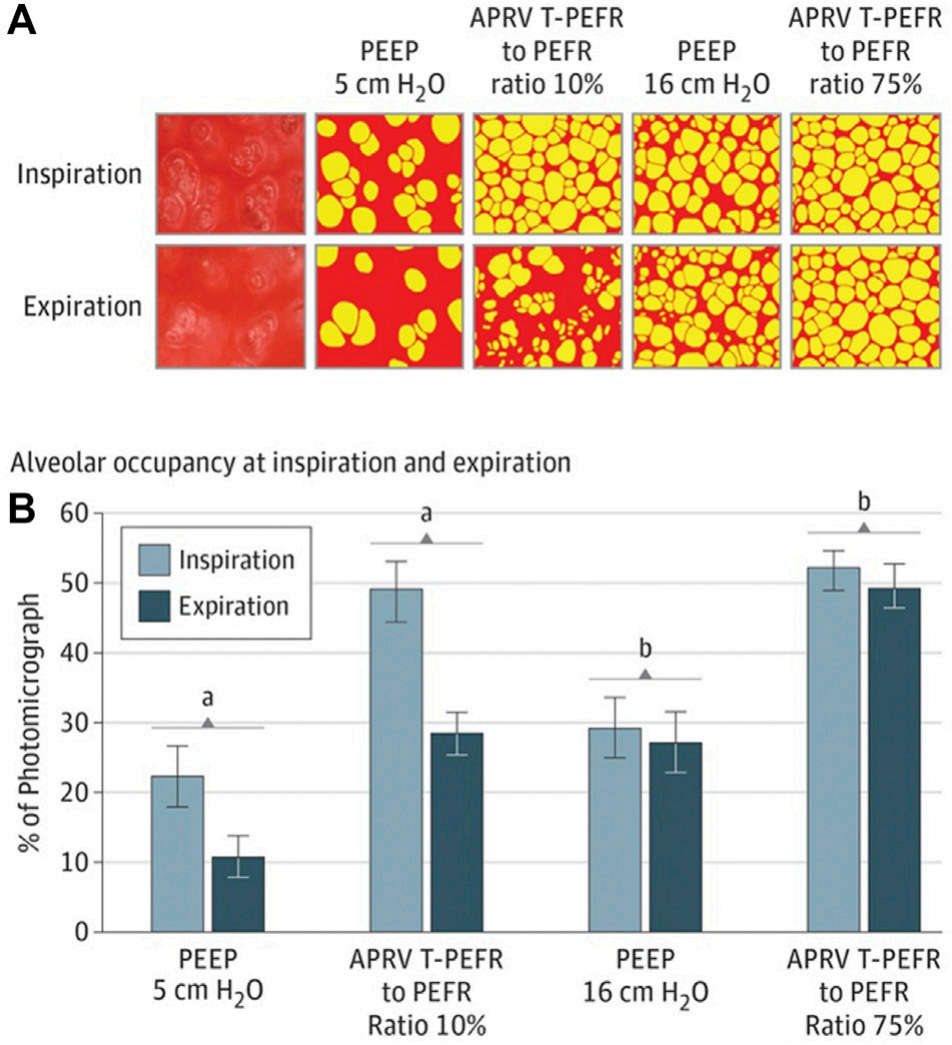
**(A)** In vivo photomicrographs at inspiration and expiration (I/E) left to right: 1) positive end-expiratory pressure (PEEP) 5 cmH_2_O; 2) airway pressure release ventilation (APRV) ratio of termination of peak expiratory flow rate (E_FT_) to peak expiratory flow rate (E_FP_) of 10%; 3) PEEP 16 cm H2O; and 4) APRV E_FT_/E_FP_ 75% (original magnification ×10). Alveoli (yellow) and nonalveolar tissue (red). **(B)**, Alveolar air space occupancy is conveyed as a percentage of the photomicrograph containing inflated alveoli (yellow in A) at I/E. Data are shown as the mean; error bars indicate standard error of the mean. A) P<.0—PEEP 5 cmH_2_O vs E_FT_/E_FP_ 10%; B) P<.05—PEEP 16 cmH_2_O vs E_FT_/E_FP_ 75. Alveolar occupancy I/E shows that APRV 75% has the greatest number of open airspaces with inspiration, which is nearly double that of PEEP 16 cmH_2_O and least loss of open airspace during exhalation resulting a less than 5% alveolar volume change between I/E. This results in the lowest micro-strain with APRV 75%.

**FIGURE 8:**
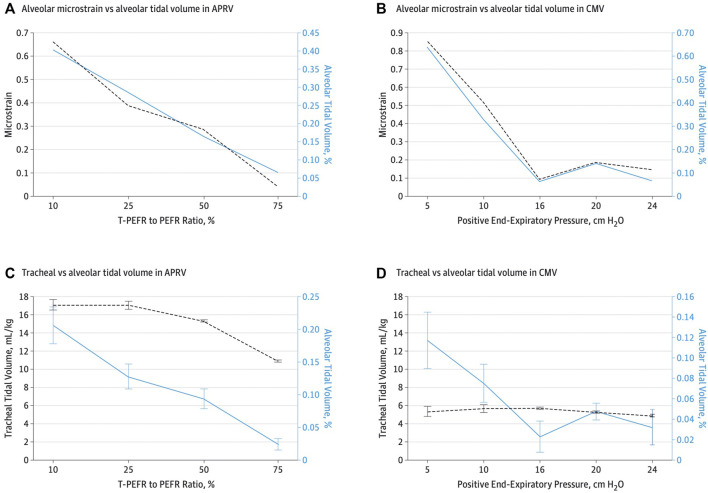
**(A,C)**—As T_Low_ is adjusted towards 75% termination of peak expiratory flow rate (E_FT_) to peak expiratory flow rate (E_FP_), alveolar tidal volume (V_T_) decreases despite tracheal volume 11 mL/kg. **(B,D)** with low V_T_ strategy, the opposite is true despite 6 ml/kg tracheal V_T_ with higher alveolar V_T_. At peep of 10 cmH2O, the alveolar V_T_ and a tracheal V_T_ of 6 ml/kg is more that 3 times higher than alveolar V_T_ with APRV 75 % despite a tracheal V_T_ of 11 ml/kg.

In an uncontrolled sepsis-induced ARDS porcine model, preemptive application of APRV using the TCAV™ method was compared to ARDSNet LV_T_ (Roy S. et al., 2013). In this model of ARDS prevention, the lung was normal and uninjured at the onset of the experiment. In the APRV group, the lung C_RS_ remained normal throughout 48-h of uncontrolled sepsis, and V_T_ maintained at 12 ml/kg yet prevented the development of ARDS or volutrauma whereas the LV_T_ group with V_T_ of 6 ml/kg developed severe ARDS. This further supports that V_T_ should be normalized to C_RS_, which was shown in the V_T_ data (Deans et al., 2005), ∆P data (Amato et al., 2015; Costa et al., 2021; Goligher et al., 2021; Raschke et al., 2021) and strenuous exercise data where V_T_ range from 36 to 40 ml/kg (Dominelli et al., 1985; Harms et al., 1998; Guenette et al., 2007; Guenette et al., 2009). With the TCAV™ method, when lung C_RS_ improves the V_T_ generally increases, which would then allow the P_High_ to be reduced and potentially the T_High_ to be extended. Additionally, in a mechanistic study with acute lung injury, APRV using TCAV™ had larger tracheal V_T_ displayed on the ventilator (macro-ventilation), yet the alveolar V_T_ (micro-ventilation) was lower than VAC with set and measured V_T_ of 6 ml/kg (Kollisch-Singule et al., 2014a). In this study, alveolar V_T_ was defined as the alveolar area change between inspiration and expiration (Figure 7). In the APRV group, area change was <5% with the T_Low_ set to 75% *E*_FT_/*E*_FP_; whereas the LV_T_ group demonstrated a 50% area change even with the most clinically used PEEP level (10 cmH_2_O) (Bellani et al., 2016) (Figure 8) suggesting this commonly used PEEP level is associated with significant atelectrauma. Further, *in-vivo* microscopy of subpleural alveoli show the T_Low_ tuned to C_RS_ (i.e., 75% *E*_FT_/*E*_FP_) stabilizes alveoli within one breath cycle halting repetitive alveolar collapse and expansion (RACE)-induced atelectrauma (Kollisch-Singule et al., 2014a). With the TCAV™ method, the passive exhalation of the T_Low_ generates the SLOPE_EF_ used to personalize V_T_ to C_RS_ (Dixon and Brodie, 1903; Rahn et al., 1946; Mead and Whittenberger, 1953; Brody 1954; Comroe 1954; Brody and Dubois, 1956; McIlroy et al., 1963; Bergman 1966; Grimby et al., 1968; Ashutosh and Keighley, 1978; Behrakis et al., 1983; Richardson et al., 1989; Baydur and Carlson, 1994; Brunner et al., 1995; Guttmann et al., 1995; Nassar et al., 2012). The SLOPE_EF_ of T_Low_ characterizes elastic recoil (E_RS_) including the chest wall and adapts to evolving lung mechanics, thereby optimizing alveolar stability and guides personalization of T_Low_, normalizing EELV and V_T_ to C_RS_ which should not be set as a fixed duration or adjusted <75% *E*_FT_/*E*_FP_ to achieve a desired V_T_.

**FIGURE 9:**
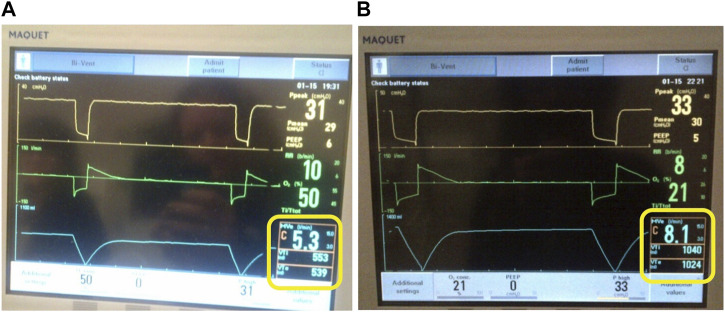
Ventilator set in the Bi-Vent (APRV) Mode. **(A)** T_Low_ set to 0.5 s and release time is 0.5 s with V_T_e 539 ml. **(B)** T_Low_ (release time) is kicking out to 1.0 s despite being set at 0.5 s with dramatically increased to V_T_e 1024 ml. This occurs in ventilators that allow pressure support (inherent trigger and trigger windows) to be added on top of the P_High_.

Finally, ventilators that can use pressure support with APRV incorporate a trigger window to attempt synchronization of inspiratory to expiratory (I:E) ratio creating an unstable T_Low_ that may randomly “kick out” beyond what is set (Figure 9). This has been shown to generate exceedingly high V_T_ where the actual T_Low_ displayed on the graphic waveform is a greater duration than the set T_Low_. The example in Figure 9 shows that despite a T_Low_ setting of 0.5 s (Figure 9A), the T_Low_ is extended to approximately 1.0 s (Figure 9B), subsequently creating a high V_T_. The video shows the spontaneous changes in the duration of the T_Low_ without any changes to the T_Low_ setting. (Supplementary Video S1). Additionally, in some variations of APRV (i.e., BiLevel on the Covidien ventilator) if the user sets T_High_ but not T_Low_, subsequent RR changes unwittingly increase T_Low_ duration resulting in a larger V_T._ This unintended consequence can be avoided by locking the T_Low_, which eliminates linking the T_Low_ with the RR, keeps the T_Low_ fixed to the intended setting and avoids inadvertently generating a larger V_T_.

## Myth #4—Airway Pressure Release Ventilation Increases Right Ventricle Afterload and Strain

Several papers warn the use of APRV leads to an increase in right ventricular (RV) afterload, worsening of pulmonary hypertension and RV dysfunction, and reduction of venous return (VR) leading to systemic hypotension (Kallet, 2011; Modrykamien et al., 2011; Chatburn et al., 2016; Chen et al., 2017). There are even claims that APRV theoretically has an increased risk of cor pulmonale (Kallet, 2011; Chatburn et al., 2016; Chen et al., 2017). Indeed, applied airway pressure can result in a reduction of VR and cardiac output (CO). However, no scientific evidence exists this occurs more frequently with APRV than any other mode as these claims suggest. Although cor pulmonale is associated with increased mortality, no study has shown this increase in mortality is linked with APRV compared to LV_T_. In fact, meta-analyses suggest the bias is towards greater survival in APRV (Lim and Litton, 2019; Zhong et al., 2020). This makes such claims implausible and uncredible leaving a basic review of physiology necessary to help navigate these misconceptions (Luecke and Pelosi, 2005). It must be realized that ventilator settings and lung-chest wall interactions have a key role in affecting the heart and these interactions may not be intuitive. Although some aspects of positive pressure may be beneficial, such as left ventricular afterload reduction with CPAP, most myths are related to RV function with inferences to systemic hypotension occurring more frequently with APRV than other ventilator modes.

Since the RV is incapable of generating significant pressure due to limitation of muscle mass, it relies on the large pressure drop across the vast highly distensible pulmonary vascular bed to limit flow resistance. The pressure drop occurs in small but numerous pulmonary vasculature, which are equally distributed between arterial and venous pulmonary circulation with the pulmonary artery having the highest resistance in the circuit (Gaar et al., 1967). Right heart loads are related to lung volume, pulmonary vascular resistance (PVR) and pleural pressure changes. Since the pulmonary circuit impedes RV output, anything affecting the lung can have an impact on right heart performance.

**FIGURE 10:**
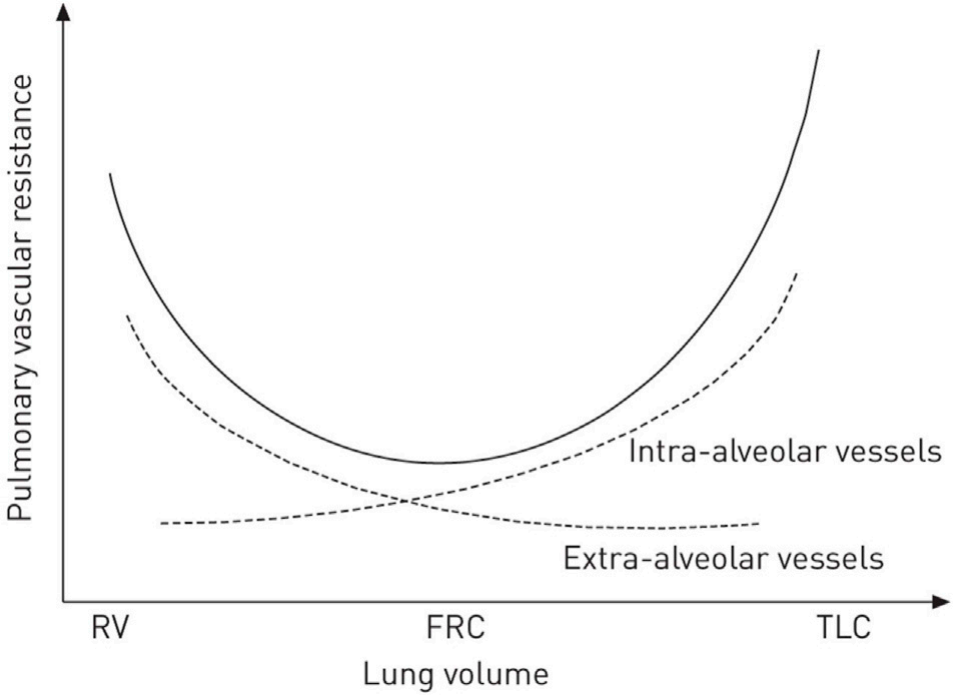
Pulmonary vascular resistance (PVR) is at its lowest at functional residual capacity (FRC). At extremes of lung volume from residual volume (RV) to total lung capacity (TLC), PVR is increased, thereby increasing RV afterload.

First, PVR and right heart load are increased at extremes of lung volume—1) residual volume (lung volume); and 2) total lung capacity (TLC) (Suresh and Shimoda, 2016) as seen in Figure 10. The lowest PVR and subsequent RV afterload is when the lung is at functional residual capacity (FRC) (Simmons et al., 1961). Many patients requiring mechanical ventilation have a loss of FRC (i.e., atelectasis) (Rahn et al., 1946; Puybasset et al., 1998; Rylander et al., 2004; Bikker et al., 2008; Bellani et al., 2011; Gonazalez-Lopez et al., 2012; Gommers, 2014; Hopkins and Sharma, 2022) and positive airway pressure to restore FRC generally results in decreased PVR and improved RV function by pulmonary artery wave-reflection (Sipmann et al., 2018) and echocardiogram (Duggan et al., 2003). Second, lung–chest wall interaction also influences hemodynamics and EELV. Similarly, this concept may also not be intuitive and goes beyond the oversimplified perception that RV load is solely a function of applied airway pressure or PEEP (Van Den Berg et al., 2001). For example, the chest wall springs out to a higher volume at the end of expiration while the lung simultaneously recoils to a lower volume with the abdominal cavity defining a boundary (diaphragm) of the chest wall and functioning as a fluid compartment rather than an elastic structure (Agostoni and Hyatt, 1973; Agostoni and Hyatt, 1986; West, 1989; Nunn, 1995; Lumb, 2010). Because of the spring out effect of the chest wall, a negative pleural pressure occurs at end-expiration even at high PEEP levels, which functionally results in the lung being suspended without any compressional forces from the chest wall at end-expiration (Stenqvist et al., 2012; Stenqvist et al., 2015; Persson et al., 2016; Persson et al., 2017). Increasing PEEP leads to lung inflation and displacement of the chest wall and diaphragm to a new pressure–volume equilibrium progressively lowering pleural pressure over subsequent breaths (Rahn et al., 1946; Katz et al., 1981; Stenqvist et al., 2012). Because right atrial pressure and VR are potentially influenced by pleural pressure, increased adaptation of the slow chest wall compartment allows EELV to increase without elevating pleural pressure (Stenqvist et al., 2012; Stenqvist et al., 2015; Persson et al., 2016; Persson et al., 2017). In fact, even RMs with high airway pressure are better tolerated hemodynamically if done incrementally rather than a sudden increase in pressure (Odenstedt et al., 2005; Santos et al., 2016). This may explain how patients with high potential for lung recruitment have less hemodynamic compromise in response to an increase in airway pressure compared to patients with non-recruitable lungs. However, data shows that ∆P (rather than PEEP per s) is associated with increased risk of cor pulmonale and the hemodynamic effect of PEEP is dependent on lung recruitability (i.e., the reduction in non-aerated lung in response to an increase in pressure) (McGuinness et al., 2020; Gazivoda et al., 2021; Hamouri et al., 2021; Rajdev et al., 2021; Udi et al., 2021).

The basic interaction between VR and positive pressure ventilation is also frequently misunderstood. Since VR is governed by the mean systemic pressure (MSP)-right atrial (RA) gradient, the application of PEEP and its impact on RA pressure would (in theory) reduce the MSP-RA gradient and decrease VR. However, many studies show the mechanism of PEEP on VR is not a reduction of the gradient as the applied pressure to the thorax is simultaneously transmitted to the abdominal compartment acting as a fluid filled compartment (Fessler et al., 1989; Nanas and Magder, 1992; Fessler et al., 1993). As a result, the pressure equally elevates the MSP, preserving the gradient for VR. Fessler et al. (1993) using MRI showed as PEEP and lung volume increases, an equal pressure point is reached compressing the vena cava as it enters the thorax from the abdomen, functioning as a starling resistor decreasing VR and impairing RV filling (Knowlton and Starling, 1912). Ultimately, lung volume is the main detriment of pleural pressure changes and can affect VR, right atrial pressure and RV afterload (O’Quinn et al., 1985).

These physical concepts of lung-heart interactions apply to all modes of ventilation. In particular, extremes of lung volume should be avoided and maintaining lung volume at FRC has the best effect on cardiopulmonary status (Figure 10). If lung volume is significantly below FRC (ie. residual volume), the airway pressure required to increase EELV will not increase heart strain; conversely if lung volume is above FRC (i.e., TLC), increased airway pressure will increase heart strain. In fact, as lung volume improves with recruitment, the size of the right heart is reduced (Duggan et al., 2003). Duggan, et al. (2003) showed that 150 min of derecruitment in rats resulted in marked dilation of the RV, paradoxical position of the interventricular septum and an underfilled left ventricle. Once the lung was recruited with an increase in applied airway pressure, there was a reduction in RV overload and improved left ventricular filling and lactate clearance. Many studies show an increase in lung volume with RMs or an appropriate increase in PEEP level improves RV function and pulmonary artery pressure (Reis Miranda et al., 2004; Reis Miranda et al., 2006; Longo et al., 2017). In general, the prevalence of cor pumonale during LV_T_ seems to increase in patients ventilated with lower PEEP levels (Boissier et al., 2013). A prospective sample of 200 patients receiving various ventilator modes, showed APRV was associated with the lowest ∆P when compared to VAC or pressure control ventilation (PCV) (Andrews et al., 2019).

To date, there have been no studies demonstrating increased hypotension or increased vasoactive use with APRV compared to any other ventilator mode whereas several studies show no difference or improved hemodynamics in APRV compared to other modes. For instance, in ARDS patients with cardiac dysfunction, APRV was shown to reduce vasoactive requirements while improving cardiac index, urine output, and lactate clearance (Kaplan et al., 2001). Additionally, a meta-analysis of seven RCTs with 405 eligible patients, showed APRV had a significantly higher mean arterial pressure on day 3 (Zhong et al., 2020) and a RCT comparing APRV to PCV in post cardiac bypass patients showed there was a significantly higher stroke volume, CO, and PaO_2_/FiO_2_ (P/F) ratio with APRV (Ge et al., 2021). More recent data showed a reduction of vasoactive support in CARDS patients managed with APRV (Joseph et al., 2020). Additionally, pediatric data includes a pediatric case series that showed APRV could safely be used in pediatric ARDS patients without significant hemodynamic deterioration (Kawaguchi et al., 2015), no difference in hemodynamic instability in pediatric patients when comparing APRV with LV_T_ (Ganesan et al., 2018) and Walsh et al. (2011) showed pulmonary blood flow, oxygen delivery and CO [in Tetralogy of Fallot group] were all significantly improved with APRV compared to PCV in children undergoing cardiac surgery. Lastly, experimental studies have shown no difference or an improvement in hemodynamics with less vasoactives and a higher MAP in APRV using the TCAV™ method compared to conventional modes including LV_T_ (Roy et al., 2012; Roy S. et al., 2013; Roy SK. et al., 2013; Emr et al., 2013; Kollisch-Singule et al., 2015; Kollisch-Singule et al., 2016b; Jain et al., 2017; Vasconcellos de Oliveira et al., 2022).

## Myth #5—it is Difficult to Control PaCO_2_ With Airway Pressure Release Ventilation

The misconception regarding inability to control partial pressure of arterial CO_2_ (PaCO_2_) leads clinicians to believe it is the ventilator mode that controls the settings and not the operator. For instance, it has been said “In APRV, some degree of CO_2_ retention is not unusual” (Modrykamien et al., 2011), “mandatory breaths in APRV are intentionally set at a lower frequency (i.e., 10 breaths/min) than for conventional modes” (Mireles Cabodevila and Kacmarek, 2016), and the RR with APRV is usually 8–12 breaths/minute (b/min) (*sic*) (Daoud et al., 2012). These claims are simply not true as there is just as much ability to control PaCO_2_ and set a higher RR in APRV as any other ventilator mode. In fact, APRV has been shown to be more efficient with PaCO_2_ removal. A review of literature specific to PaCO_2_ clearance with APRV spanning 25 years demonstrates APRV is associated with lower PaCO_2_ when minute ventilation (MVe) is matched or a similar PaCO_2_ with less MVe (Stock et al., 1987; Valentine et al., 1991; Smith and Smith, 1995; Maung et al., 2011). In other words, the volume of CO_2_ (VCO_2_) per liter of exhaled V_T_ is greater in APRV as compared to conventional ventilation (Bratzke et al., 1998). In addition, PaCO_2_ depends on two phenomena: 1) physiological dead-space *per se;* and 2) the increased PaCO_2_ seen in the case of high shunt fraction particularly when there is an increased gradient between the mixed-venous blood and PaCO_2_. Increasing the inspiratory time allows more time for diffusive exchange of PaCO_2_ where expiration begins when alveolar CO_2_ (PACO_2_) is close to equilibrium with mixed venous blood. Conversely, with a brief inspiratory time, expiration begins when PACO_2_ is at its nadir. Physiologic data demonstrate optimizing diffusive and convective gas exchange [bulk flow of exhaled gas into the environment] increases ventilation efficiency, thus lowering MVe requirements for equivalent PaCO_2_ clearance (Haycroft and Edie, 1891; Knelson et al., 1970; Engel et al., 1973; Fukuchi et al., 1976; Fuleihan et al., 1976; Fredberg, 1980; Valentine et al., 1991; Falkenhain et al., 1992; Smith and Smith, 1995; Mercat et al., 2001; Tsuda et al., 2011; Aboab et al., 2012). The concept that alveolar recruitment and derecruitment is time-dependent is often overlooked by clinicians. Although there is variability in alveolar recruitability among ARDS patients, time remains a critical element of distal airspace reopening and closure (Allen et al., 2002; Allen and Bates, 2004; Allen et al., 2005; Albert et al., 2009).

In addition to controlling the RR, the T_High_ promotes gradual time-dependent alveolar recruitment throughout the lung, thereby reducing shunt fraction and increasing lung surface area for exchange of PaCO_2_ based on Fick’s Laws of Diffusion (Fick 1855; Wagner 1977). However, this does not imply that all patients, particularly those with significant lung dysfunction (i.e., ARDS) should have APRV initiated at a rate of 8–12 b/min (ie., T_High_ 4–6 s). Rather, the T_High_ should be adjusted to provide adequate ventilation and PaCO_2_ for a given degree of pulmonary dysfunction. As surface area increases and alveolar stability improves, diffusive gas exchange increases and need for convective gas exchange (i.e., RR) decreases. Progressively, ventilation becomes more efficient over time (12–36 h) enabling an appropriate T_High_ increase. Correcting hypercarbia with T_Low_ manipulations to generate a larger V_T_ may briefly improve PaCO_2_, but reduction in diffusive surface area from lung volume loss occurs, ultimately sacrificing alveolar stability and subsequently the mode is blamed for the high V_T_ and hypercarbia simultaneously.

In studies criticizing the inability of APRV to manage PaCO_2_ (Batchinsky et al., 2011; Ibarra-Estrada et al., 2022), the T_Low_ was increased [*E*_FT_/*E*_FP_ <75%] to adjust for hypercarbia, which resulted in a V_T_ increase that has been shown to subsequently increase alveolar collapse, worsen alveolar instability and heterogeneity, micro-strain and stress risers throughout the lung (Kollische-Singule et al., 2014a; Kollische-Singule et al., 2014b; Kollische-Singule et al., 2016a; Jain et al., 2017). Consequently, when the T_Low_ is adjusted to *E*_FT_/*E*_FP_ <75%, alveolar collapse and instability ensues, ultimately resulting in further hypercarbia. In addition, rather than adjusting the T_High_ to increase the RR, these studies used a much lower RR in the APRV group compared to conventional modes (Batchinsky et al., 2011; Ibarra-Estrada et al., 2022). Conversely, in a large study of 411 patients in a burn unit using APRV, pH and PaCO_2_ were maintained in the normal range with improved P/F ratios (Foster et al., 2021) and Maxwell, et al. (2010) reports the most interesting finding in their study was the LV_T_ group had a higher PaCO_2_ than the APRV group despite a significantly higher MVe.

If patients with pulmonary dysfunction receiving APRV are treated the same as stable mechanically ventilated patients in terms of convective ventilation (i.e., RR 8–12 with T_High_ 4–6 s) hypercarbia would be expected. This was shown in a recent study of CARDS (Ibarra-Estrada et al., 2022) where more patients in the APRV group had transient (≤24 h) episodes of severe hypercapnia (42% vs 15%; *p* = 0.009) but were not associated with hemodynamic changes. However, the APRV group was managed with a T_High_ 4–6 s, which translated into ∼10–12 b/min resulting in a significantly lower RR as compared with LV_T_ group (*p* < 0.001). It is important to note these patients had moderate to severe ARDS P/F ratios (per Berlin criteria) (ARDS Definition Task Force, 2012) from COVID, a pulmonary pathology with high dead-space fraction (Morales-Quinteroset al., 2021).

## Myth #6—Airway Pressure Release Ventilation is the Same as Inverse Ratio Pressure Control

**FIGURE 11:**
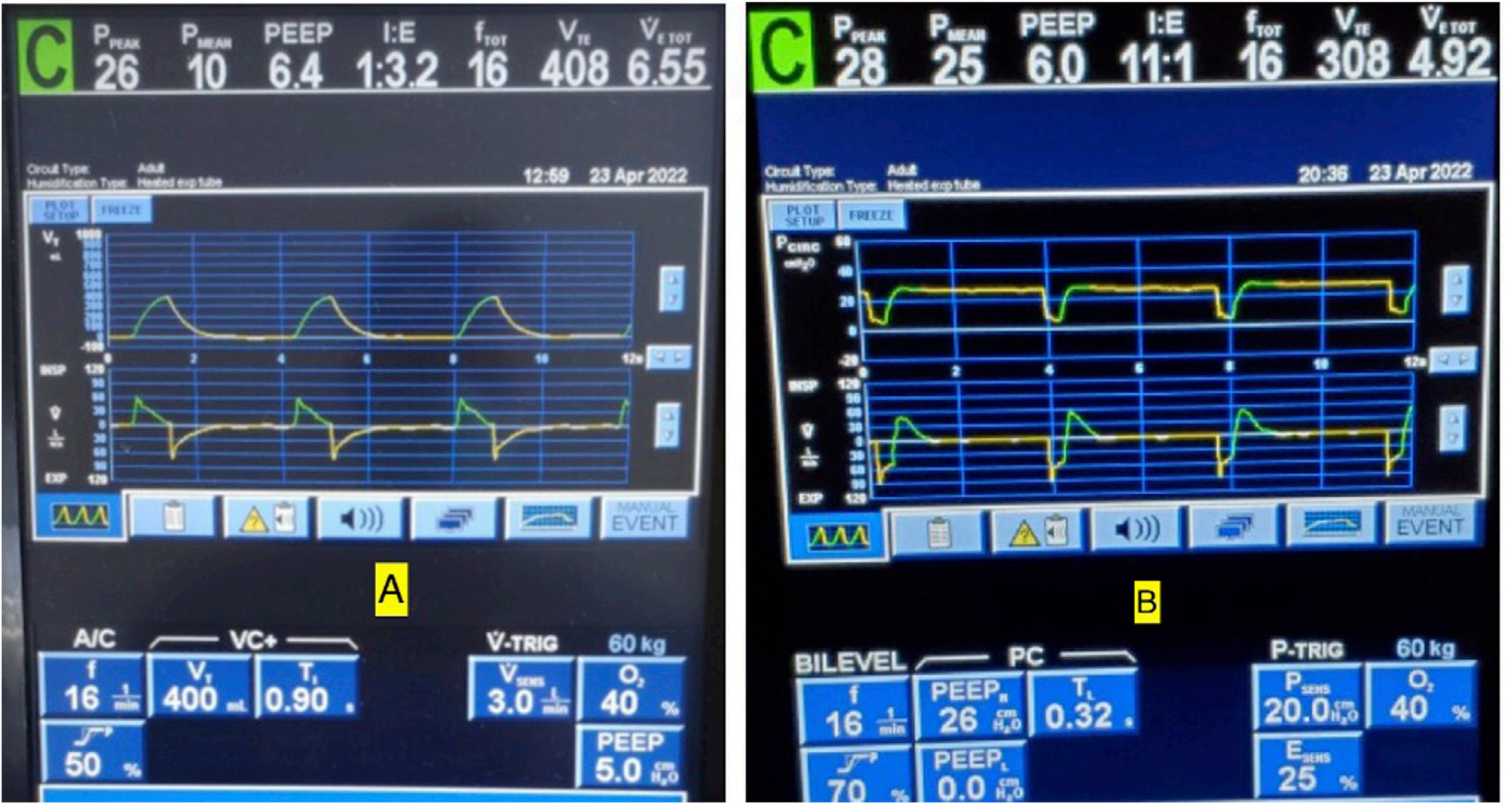
**(A)** Conventional volume assist control (VAC) mode with a set respiratory rate (RR) of 16 and inspiratory to expiratory (I:E) ratio of 1:3.2. **(B)** Same patient transitioned to BiLevel (APRV) with same rate and T_Low_ set to 0.32 s to terminate at 75% of peak expiratory flow rate (*E*_FT_/*E*_FP_) yields an I:E ratio of 11:1. Note also that at *E*_FT_/*E*_FP_ 75%, the tidal volumes decreased from 408 to 308 ml to match current C_RS._

Several papers remark that APRV is functionally the same and indistinguishable from inverse ratio PCV (IR-PCV) in the absence of spontaneous breathing (Dries and Marini, 2009; Esan et al., 2010; Kallet 2011; Mireles Cabodevila and Kacmarek, 2016). Although it is true that both modes share similarities with settings that control pressure and time, there are key differences that are often overlooked. The first key difference is the inspiratory and expiratory times in APRV are controlled directly, independently and precisely, whereas I:E ratios of time are utilized in IR-PVC with the expiratory phase a “by-product” resulting indirectly from a set inspiratory time and RR. Comparable to the RR setting in IR-PCV, APRV uses the T_High_ to control RR where counterintuitively a decrease in T_High_ increases RR and an increase in T_High_ decreases RR. In addition, like the inspiratory time in IR-PCV, the T_High_ regulates the duration of the P_High_ creating a CPAP Phase to promote gradual expansion of collapsed alveoli (Syring et al., 2007; Boehme et al., 2015). However, because of the brief T_Low_ duration, APRV with an equal RR typically has a much higher I:E than is possible with IR-PCV on most ICU ventilators (Figure 11), which becomes progressively more limited with IR-PCV as the set RR increases. Figure 11 shows conventional VAC (11A) with a set RR of 16 and I:E ratio of 1:3.2 transitioned to APRV (BiLevel on the Covidien) (11B) with same RR and the T_Low_ set to 0.32 s to achieve 75% E_*FT*_/E_*FP*_ yielding an I:E ratio of 11:1. Subsequently, the V_T_ decreased from 408 to 308 ml (Nieman et al., 2020a; Nieman et al., 2020b).

The second key difference is that unlike IR-PCV, PEEP is not typically set with APRV because EELV is controlled with *time* (T_Low_) rather than *pressure* (P_Low_). Although studies show a P_Low_ in APRV may be set at any level, it is generally set at 0 cmH_2_O when the T_Low_ is used as the controller of EELV (Habashi, 2005; Habashi et al., 2022). In fact, we have shown that personalizing the T_Low_ to *E*_FT_/*E*_FP_ 75% in acute restrictive lung disease (i.e., increased E_RS_) allows for quick stabilization of alveoli by halting alveolar collapse, loss of EELV and RACE-induced atelectrauma (Roy et al., 2012; Roy S. et al., 2013; Roy SK. et al., 2013; Andrews et al., 2013b; Emr et al., 2013; Kollisch-Singule et al., 2014a; Kollisch-Singule et al., 2014b; Kollisch-Singule et al., 2015; Kollisch-Singule et al., 2016a; Kollisch-Singule et al., 2016b; Kollisch-Singule et al., 2019; Smith et al., 2015; Jain et al., 2017; Silva et al., 2018; Bates et al., 2020; de Magalhã;es et al., 2021; Vasconcellos de Oliveira et al., 2022). Additionally, when the P_Low_ is set to 0 cmH_2_O, the SLOPE_EF_ is used to analyze the expiratory recoil forces, which allows personalization with fine-tuning of the T_Low_ to a patient’s lung mechanics. This allows the clinician to adjust T_Low_ for changes in EELV and C_RS_, based on the SLOPE_EF_. This real-time breath to breath bedside monitoring of respiratory mechanics is not possible with IR-PCV as the PEEP valve attenuates the recoil force distorting the SLOPE_EF_, which no longer reflects E_RS_ of a passive exhalation (Dixon and Brodie, 1903; Rahn et al., 1946; Mead and Whittenberger, 1953; Brody 1954; Comroe 1954; Brody and Dubois, 1956; McIlroy et al., 1963; Bergman 1966; Grimby et al., 1968; Ashutosh and Keighley, 1978; Behrakis et al., 1983; Richardson et al., 1989; Baydur and Carlson, 1994; Brunner et al., 1995; Guttmann et al., 1995; Nassar et al., 2012).

Lastly, the name of the ventilator mode and what is configurable by the user varies among ventilator brands. For instance, APRV is often confused with variants of APRV (i.e. BiPAP, Bilevel) as manufacturers have their own branding of the mode APRV such as: Bi-Vent/APRV (Servo/Maquet), BiLevel/PC (Puritan Bennett/Covidien), APRV/BiPhasic (Avea/CareFusion), and APRV/PC-APRV (Dräger) to name a few. The crucial element when selecting the APRV mode, is the ability to set and adjust T_High_ and T_Low_ independently and precisely.

## Myth #7—Airway Pressure Release Ventilation Creates Unsafe Auto-Peep

It has been the view of some that APRV leads to uncontrollable and even unsafe auto-PEEP (Dries and Marini, 2009; Modrikyniem et al., 2011; Daoud et al., 2012). Although a common statement, no data exists to support uncontrolled auto-PEEP and dynamic hyperinflation (DHI) occurs solely or with greater frequency in APRV (i.e., CPAP with release) than any other ventilator mode. Both terms and perception about auto-PEEP as it applies to APRV are assumed to equal DHI, which can cause barotrauma and hemodynamic instability and—by definition - increases over time. However, retaining static EELV should not be conflated to be equivalent to DHI and in fact, static lung volume with CPAP (i.e., without release) can decrease DHI in COPD (Petrof et al., 1990; Fessler et al., 1995; O’Donahuhe et al., 2002; Lopes et al., 2011). This opinion about auto-PEEP arises because APRV does not conform to the canonical practice of a set PEEP. Rather, in the TCAV™ method the T_Low_ prevents airway closure and retains EELV with brief, precise time control personalized to an individual’s respiratory system mechanics [recoil force]. As EELV is a function of E_RS_ and the PEEP-volume, which is proportional to FRC and determined by T_Low_ duration and given that T_Low_ is adjusted based on a fixed percentage of the expiratory flow, which is an integral of volume, rather than a fixed or arbitrary time, volume displacement and EELV are therefore controlled directly.

Normally, lung volume at end-expiration approximates relaxation volume of the respiratory system. This defines FRC where the recoil forces of the lung towards the hilum are neutralized by outward forces of the chest wall and functions to maintain stable gas exchange, minimize elastic work of breathing (WOB) and optimize cardiopulmonary function (Rahn et al., 1946). Loss of FRC is common in hospitalized patients receiving mechanical ventilation (termed EELV) (Puybasset et al., 1998; Rylander et al., 2004; Bikker et al., 2008; Bellani et al., 2011; Albert, 2012; Gonazalez-Lopez et al., 2012; Gommers, 2014; Albert, 2022; Hopkins and Sharma, 2022) and is magnified in ARDS where the role of static EELV is not only essential for cardiopulmonary benefits but may improve effectiveness of lung protective strategies as it minimizes lung strain, which can be high despite LV_T_ strategy (Chiumello et al., 2008; Gonzalez-Lopez et al., 2012; Xie et al., 2017).

Although set PEEP is intended to maintain or increase EELV by producing an expiratory retard, this view of creating auto-PEEP portends that only in APRV is the increase in EELV uncontrollable. Since adequate EELV during mechanical ventilation is necessary in protective ventilation, a reasonable question remains whether to use a set pressure (PEEP) to *indirectly* maintain EELV or guiding flow-time (T_Low_) to *directly* retain and control EELV, as volume is an integral of flow. Since both PEEP and T_Low_ can maintain EELV, the key distinction is between static inflation (wanted) vs DHI (unwanted). The general concern for auto-PEEP, “air trapping” and DHI with APRV seems to be a reaction to the brief expiratory release time (T_Low_). However, the role of expiratory time during mechanical ventilation has little impact on relieving DHI even in COPD (Leatherman et al., 2004; Ku, 2016; Natalini et al., 2016). Leatherman et al. (2004) noted extending expiratory time to >7 s did not significantly change DHI even in status asthmatics patients. Similarly in 186 patients with air flow limitations/obstructive lung disease, Natalini et al. (2016) states “Surprisingly, we observed that in our sample of mechanically ventilated subjects, the variables that characterized the breathing pattern (f, TE, VT, and minute ventilation) appeared to have a marginal role in auto-PEEP” and “It appears that even in patients with airflow limitations, Auto-PEEP can be more effectively reduced by acting primarily on modifiable characteristics of the patient, whereas manipulation of the breathing pattern might only have a negligible effect on the overall auto-PEEP value.”

The equal pressure point contributes to increased airway resistance in addition to elastic recoil producing airflow limitations and delaying lung emptying allowing the next inspiratory effort/breath to occur before static equilibrium volume is reached resulting in DHI (Voets and Van Helvoort, 2013). Additionally, in patients with airflow limitations EELV may exceed predicted FRC (Kimball et al., 1982; Pepe and Marini, 1982). Despite being well described in the literature, the incidence of auto-PEEP remains unknown; however, most cases of DHI occur in patients with airflow limitations even without mechanical ventilation or typically receiving conventional ventilation (Wright and Gong, 1990; O’Donnell and Laveneziana, 2006). Whereas as “low level” auto-PEEP has been described with LV_T_ (Marini et al., 1985; de Durante et al., 2002; Patroniti and Pesenti, 2003). Bergman (1972) first described progressive air trapping using the term DHI that was induced by increasing RR up to 66 b/min coupled with increase in V_T_ up to 1 L in seven anesthetized patients. Subsequently, Pepe and Marini (1982) described the “auto-PEEP effect” in a case series describing DHI in three patients, two with known COPD and one with active bronchospasms using 11–12 ml/kg V_T_ with VAC. Because patients with COPD exhibit a decrease rate of lung emptying toward the end of expiration due to an increase in R_AW_ and are at greatest risk for DHI, a set PEEP is used to decrease R_AW_ and as a result DHI. This set PEEP results in a faster and more uniform rate of lung emptying (Kondili et al., 2004), which seems to be beneficial in decreasing DHI and may improve ventilator triggering (Chao et al., 1997). Likewise, in patients with ARDS the respiratory system deflation rate progressively decreases due to a considerable increase in expiratory resistance at low lung volume (Koutsoukou et al., 2000; Kondili et al., 2002) as airway caliber decreases during lung volume loss (Wilson, et al., 1993). Thus, application of PEEP in ARDS decreases the expiratory resistance similar to that seen in COPD patients and results in a relatively constant and fast rate of lung emptying (Koutsoukou et al., 2000; Kondili et al., 2002). Additionally, lung ultra-structure data shows PEEP dilates ducts as a possible mechanism of decreasing R_AW_ (Kollisch-Singule et al., 2014b).

Since PEEP decreases R_AW_ in COPD and ARDS, increasing lung emptying may be beneficial to reduce DHI in COPD where E_RS_ is low (i.e., low recoil force); however, when E_RS_ is high (i.e., ARDS) the lung may degas rapidly promoting atelectrauma. Thus, patients with high E_RS_ accommodate less inspired lung volume and maintain high recoil forces and in the absence of significant airflow limitations make DHI less likely (Gottfried, et al., 1985; Gottfried, 1991; Marini 2011). For instance, in a saline-lavage rabbit model cyclical lung recruitment was assessed with a fast PaO_2_ probe comparing brief exhalation time (TExp) (0.83 s) and low PEEP (3 cmH_2_O) to a prolonged TExp (2.9 s) and high PEEP (14cmH_2_O) (Syring et al., 2007). Results showed compared to the low PEEP/brief TExp group, the high PEEP/prolonged TExp group experienced more cyclical recruitment (P 0.001). Furthermore, the low PEEP/brief TExp did not generate intrinsic PEEP (PEEPi). The authors summarize “Prevention of end-expiratory derecruitment without PEEPi suggests another mechanism, distinct from PEEPi, plays a role in the dynamic behavior of atelectasis.” In addition, CO was increased on average 13% in the brief TExp compared with the high PEEP group (P 0.001), as was mixed venous saturation (P 0.001). In a lavage model of ARDS in juvenile pigs Boehme et al. (2015) found a prolonged inspiratory phase leads to higher average PaO_2_ while the shortened Texp reduces tidal oscillations in PaO_2_ suggesting a reduction in cyclic recruitment - derecruitment (c-R/D) with brief Texp. Shortening the Texp with inverse ratio ventilation (IRV) reduced the time available to derecruit, resulting in more average recruitment. Using electrical impedance tomography, as the I:E increased from 1:4 to 4:1 changes in regional ventilation occurred producing a redistribution from nondependent toward dependent lung regions. Boehme et al. (2015) also found negligible intrinsic PEEP as the Texp decreased in all settings. The authors conclude “Time constants for recruitment and derecruitment, and regional ventilation distribution, reflect these findings and highlight the time dependency of cyclic recruitment and derecruitment” (Boehme et al., 2015).

Although EELV is traditionally managed with PEEP, it remains unclear what PEEP level prevents airway closure in a given patient at a given time (Kalenka et al., 2016). Although increasing PEEP shows a linear correlation with oxygenation and is commonly used as a surrogate of recruitment, it remains a poor marker of alveolar stability as seen with *in-vivo* microscopy (Andrews et al., 2015). Decremental PEEP studies show the loss of EELV at each level of PEEP reduction making one PEEP level difficult to control time dependent lung behavior (Maggiore et al., 2001; Sahetya et al., 2017; Bates and Smith, 2018; Baumgardner 2019; Broche et al., 2019; Scaramuzzo et al., 2019). Data on the effect of PEEP on lung micro-architecture suggest PEEP primarily causes ductal dilatation rather than preventing alveolar collapse and increases alveolar heterogeneity (Kollisch-Singule et al., 2014b; Kollisch-Singule et al., 2016a) (Figure 5). In fact, the PEEP-FiO_2_ scale has recently been challenged as dangerous for CARDS patients (Gattinoni et al., 2020a, 2020b; Tsolaki et al., 2020; Barthélémy et al., 2021; Ceruti et al., 2021).

**FIGURE 12:**
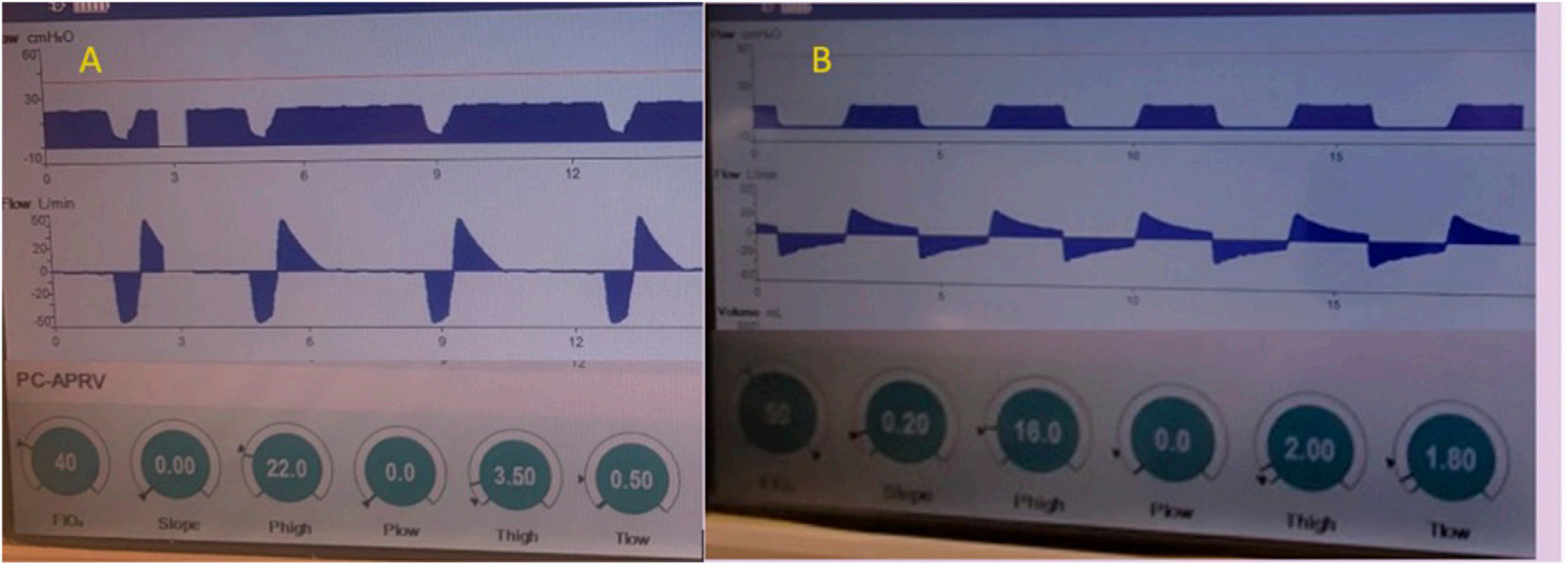
Passive exhalation to determine lung mechanics in APRV - The Time Controlled Adaptive Ventilation (TCAV^TM^) method of Airway Pressure Release Ventilation (APRV) uses the slope of the expiratory flow curve of passive exhalation to determine respiratory mechanics. Example **(A)** (left) is a patient with high elastance of the respiratory system (E_RS_) denoted by the expiratory flow rate >50 liters/minute and the acute slope deceleration angle. The slope deceleration is affected by inspiratory lung volume and downstream resistance (native and artificial airways and P_Low_ >0 cmH_2_O). Changes in E_RS_ (i.e., recoil force per unit of volume) or increase in airway resistance (airflow limitations) alters peak expiratory flow (E_FP_) and slope angle. The T_Low_ with high E_RS_ is adjusted to terminate the expiratory flow (E_*FT*_) at 75% of the peak expiratory flow (E_*FP*_). End-expiratory lung volume (EELV) is controlled through precise and personalized adjustment of flow-time as an integral of volume. Because personalization of the T_Low_ is adjusted based on elastic recoil of the lung and E_RS_, it should not be adjusted to achieve tidal volume (V_T_) or control PaCO_2_. The 75 E_*FT*_/E_*FP*_ has be calibrated experimentally, validated clinically, and shown to optimize EELV, prevent airway closure and lower lung strain in lungs with normal to increased E_RS_. Example **(B)** (right) is a patient with low E_RS_, low recoil forces and high resistance denoted by the expiratory flow rate <20 liters/minute and the less acute slope deceleration angle where the T_Low_ is adjusted to achieve 25% of E_*FT*_/E_*FP*_, which has been calibrated to decrease alveolar heterogeneity, lung inflammation, edema, and gene expression of biological markers related to ventilator induced lung injury and improve right ventricular performance by personalizing a COPD model.

**FIGURE 13:**
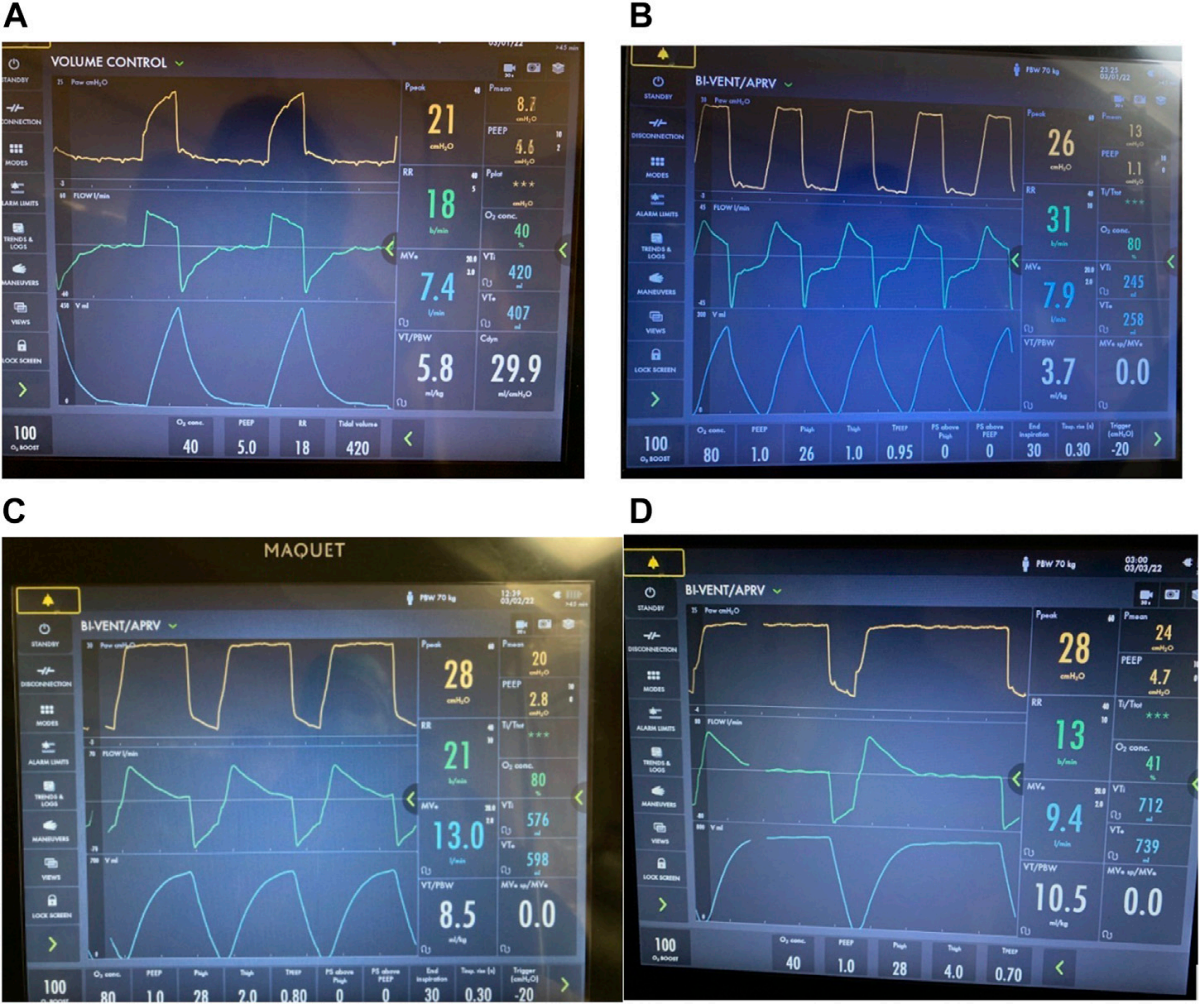
T_Low_ setting in patient with acute bronchospasm (status asthmaticus). Bedside monitoring of airflow limitations with real-time T_Low_ adjustments with airway pressure release ventilation (APRV) BI-VENT in a patient with active bronchospasm **(A)** Volume Control mode where intrinsic dynamic (Dyn) positive end expiratory pressure (PEEP) is not seen in the expiratory flow waveform **(B)** Mode changed to BI-VENT/APRV with peak expiratory flow rate (*E*_FP_) measured -20 L/min, which is consistent with severe airflow limitation. Note, *E*_FP_ is measured at onset of deceleration and not artifact from immediate loss circuit gas compression. T_Low_ is adjusted to 0.95 s targeting termination of flow rate (*E*_FT_) >25% to <50% for patients with airflow limitations. **(C)** Resolving acute bronchospasm, *E*_FP_ is nearly 70 l/min allowing T_Low_ to be decreased to 0.8 seconds while continuing to target *E*_FT_ */E*_FP_ >25% to <50%. **(D)** Continued improvement of bronchospasm where *E*_FP_ is nearly 80 l/min allowing T_Low_ to be decreased to 0.7 s while continuing to target *E*_FT_ */E*_FP_ >25% to <50%. Progressive increase in tidal volume and minute ventilation allows gradual reduction of P_High_ (not shown). Note, this ventilator does not allow a P_Low_ of 0 cmH_2_O with 1 cmH_2_O the lowest setting possible.

Alternatively, the T_Low_ set to the prevailing time constants (Bates et al., 2020) demonstrated that APRV with the TCAV™ method increases alveolar stability, decreases micro-strain and alveolar heterogeneity and normalizes the airspace with less ductal dilation than PEEP (Kollisch-Singule et al., 2014a; Kollisch-Singule et al., 2014b; Kollisch-Singule et al., 2016a) (Figures 4–7). With the TCAV™ method, the T_Low_ is adjusted to target *E*_FT_/*E*_FP_ 75% in normal to high E_RS_ (i.e., ARDS) and <50%–25% for patients with low E_RS_ (i.e., COPD, asthma) (Figure 12). Lastly, analyzing the SLOPE_EF_ with the TCAV™ method provides real-time assessment of respiratory mechanics as a patient’s disease process evolves rather than the arbitrary PEEP selection or attempts to use oxygenation as a marker of alveolar stability and a surrogate for low lung strain (Andrews et al., 2015). In APRV with a P_Low_ set to 0 cmH_2_O, air flow limitations and changes in EELV are seen in real-time with changes in E_RS_ or resistance including experimental models of COPD (Vasconcellos de Oliveira et al., 2022). Figure 13 shows the evolution of the T_Low_ in a patient with acute bronchospasm (status asthmaticus), which was captured with real-time bedside monitoring of airflow limitations and corresponding T_Low_ adjustments (Figure 13). When acceptable levels of spontaneous breathing occur and because the release phase in APRV-TCAV™ is so brief, there are three major implications: 1) spontaneous breaths occur primarily during the CPAP Phase preserving neural inspiratory time; 2) CPAP in patients with airflow limitations is associated with a decrease in DHI allowing patients to defend their lung volume making uncontrolled DHI unlikely (Petrof et al., 1990; Fessler et al., 1995; O’Donahuhe et al., 2002; Lopes et al., 2011); and 3) the active exhalation valve compared to a closed expiratory valve during the inspiratory phase allows patients to exhale beyond the set release frequency and gain an inspiratory assistance by using abdominal expiratory muscles (Torres et al., 1993).

## Myth #8—A P_LOW_ of 0 CMH_2_O Leads to Injury and Alveolar Collapse

Although which PEEP level is protective remains undefined, it has been suggested the abrupt transition from P_High_ to a P_Low_ of 0 cmH_2_O is uncontrolled in APRV creating potential for mechanical injury, which is otherwise protected by PEEP (Neumann et al., 2002; Dries and Marini, 2009) and a P_Low_ of 0 cmH_2_O allows for alveolar collapse even with a brief T_Low_ (Myers and Macintyre, 2007; Modrikyniem et al., 2011; Daoud et al., 2012). Fundamentally, when using the TCAV™ method of APRV the P_Low_ is set to 0 cmH_2_O because time [rather than pressure] is used to control EELV. Additionally, a P_Low_ >0 cmH_2_O alters the flow-time course of passive exhalation, thus dampening the recoil force where the SLOPE_EF_ no longer represents mechanics of the respiratory system.

Like PEEP, there is no consensus of the P_Low_ setting in APRV. However, studies have shown that APRV a P_Low_ 0 cmH_2_O and T_Low_ set to *E*_FT_/*E*_FP_ 75% maintains EELV, prevents end expiratory airspace collapse, produces lowest micro-strain on distal air spaces (alveoli and ducts) (Figure 4), minimizes ductal dilatation (Figure 5) and restores alveolar homogeneity after heterogenous lung injury compared to LV_T_ with PEEP up to 24 cmH_2_O (Figure 6) (Kollisch-Singule et al., 2014a, Kollisch-Singule et al., 2014b, Kollisch-Singule et al., 2016a, Kollisch-Singule et al., 2016b showed in a model of acute lung injury alveolar area change between inspiration and expiration was <5% in the APRV group with a P_Low_ of 0 cmH_2_O and T_Low_ set to *E*_FT_/*E*_FP_ 75%, mimicking the area change of uninjured lung; whereas the LV_T_ group with the most commonly clinically used PEEP of 10 cmH_2_O (Bellani et al., 2016) demonstrated a 50% area change between inspiration and expiration suggesting a 10-fold greater RACE-induced atelectrauma (Figure 8). In addition, to determine APRV efficacy, a translational model comparing APRV with LV_T_ showed the APRV group with T_Low_ set to 75% *E*_FT_/*E*_FP_ and P_Low_ 0 cmH_2_O did not produce lung injury by P/F ratio, histology, or inflammatory markers, whereas the LV_T_ group developed ARDS in all animals by P/F ratio, histology, and inflammatory markers (Roy et al., 2012; Roy S. et al., 2013; Roy SK. et al., 2013; Silva et al., 2018; de Magalhã;es et al., 2021). Further, an observational study of ARDS prevention looked at 231 patients set with an APRV protocol using T_Low_ 75% *E*_FT_/*E*_FP_ and P_Low_ 0 cmH_2_O (Andrews et al., 2013b) and did not show a higher ARDS rate or mortality as would be assumed if using T_Low_ 75% *E*_FT_/*E*_FP_ and P_Low_ 0 cmH_2_O could not limit collapse, subsequently worsening lung injury.

It has also been claimed that a P_Low_ of 0 cmH_2_O does not increase the *E*_FP_ (Zhou and Chatburn, 2012). However, this is based on data generated from a simulator model, which is unable to quantify the viscoelastic tissue behavior of the lung and chest wall, where it was speculated *E*_FP_ would remain unchanged with no delay when comparing P_Low_ settings. To achieve their goal, the P_High_ was increased with each increase in P_Low_ to maintain a ∆ 25 cmH_2_O. This of course does not represent the clinical application of APRV and would be analogous to increasing the inspiratory pressure in PCV each time a PEEP increase is made. It is not surprising their results showed an increase in *E*_FP_ with each increase in P_High_ and simultaneous increase in P_Low_ as the increased recoil in a single compartment model would be expected. However, this same concept was tested in 20 patients where only the P_Low_ was increased and not the P_High_ (Madden et al., 2016), reflecting standard clinical practice when using the T_Low_ to control EELV and showed a progressive decrease in the *E*_PF_ as the P_Low_ was sequentially increased >0 cmH_2_O.

**FIGURE 14:**
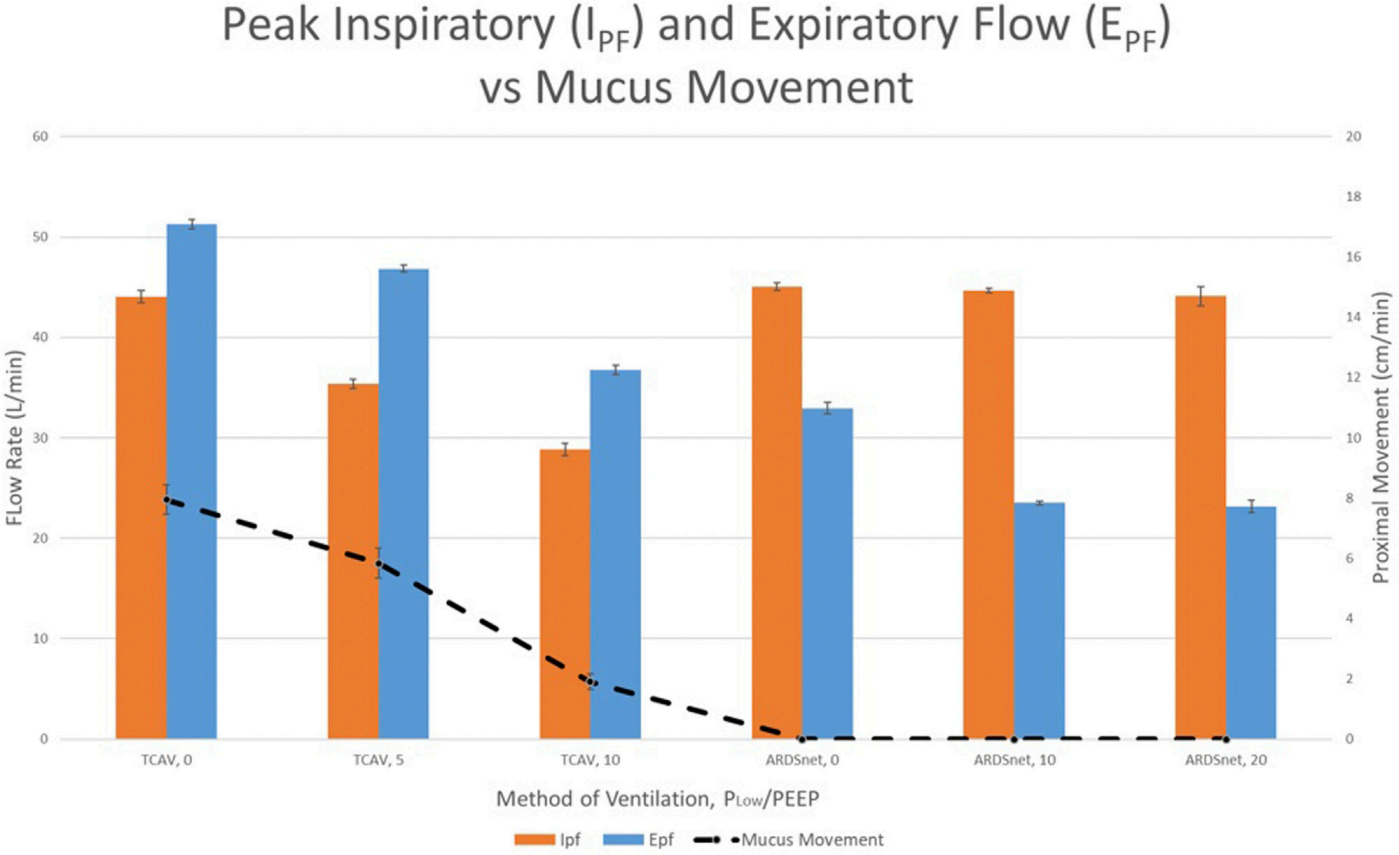
Peak inspiratory flow (I_PF_), peak expiratory flow (E_PF_), and proximal mucus movement for experimental groups comparing Airway Pressure Release Ventilation (APRV) and Low Tidal Volume (LV_T_). Orange and blue colored bars demonstrate the I_PF_ and E_PF_ respectively (left vertical axis). Proximal mucus movement is denoted by the dotted line connecting data points (vertical axis). TCAV protocol groups 1, 2, and 3 used APRV with varying P_Low_ (standard APRV-TCAV^TM^ with 0 cmH_2_O) and 5 and 10 cmH_2_O, respectively. The ARDSNet LV_T_ groups 4, 5, and 6 varied positive end expiratory pressure (PEEP) settings of 0, 10 and 20 cmH_2_O, respectively.

Because expiratory flow rates are critical for secretion removal, Mahajan et al. (2019) further validated setting a P_Low_ of 0 cmH_2_O in a model of preserved pig lungs fitted with an endotracheal tube. Multiple combinations of peak inspiratory and *E*_FP_ rates were used to compare APRV (TCAV™ method) with LV_T_ (ARDSnet protocol). The P_High_/Pplat was set equally in both groups. In the APRV group, only the P_Low_ was adjusted from 0 to 5 to 10 cmH_2_O incrementally and in the LV_T_ group, the PEEP was adjusted from 5 to 10 to 20 cmH_2_O incrementally. As the P_Low_ was increased, both *E*_PF_ and mucus movement decreased, which is important as studies suggest clearance of mucus is facilitated with increased *E*_PF_ (Kim et al., 1987; Dennesen, et al., 2003; de Prost et al., 2007; Powell et al., 2018). The APRV-TCAV™ group resulted in the greatest proximal mucus movement compared to no mucus movement in the LV_T_ group at any PEEP level as seen in Figure 14. Further, in a study comparing APRV-TCAV™ with VAC in experimental pneumonia (de Magalhã;es et al., 2021), APRV-TCAV™ was associated with less lung damage, less bacteremia and reduced gene expression of mediators associated with inflammation.

To summarize, when using the TCAV™ method of APRV setting a P_Low_ is not necessary and varied P_Low_ levels would be just as arbitrary as varied PEEP levels, which continues to be unsettled and may remain so for the foreseeable future. However, all experimental models show less injury or complete prevention of lung injury when using a P_Low_ 0 cmH_2_O with a T_Low_ 75% *E*_FT_/*E*_FP_ (Roy et al., 2012; Roy S. et al., 2013; Roy SK. et al., 2013; Kollisch-Singule et al., 2014a; Kollisch-Singule et al., 2014b; Kollisch-Singule et al., 2016a; Kollisch-Singule et al., 2016b; Silva et al., 2018; de Magalhã;es et al., 2021). Clinically, trials show APRV with P_Low_ 0 cmH_2_O with a T_Low_ 75% *E*_FT_/*E*_FP_ is not inferior to LV_T_, which would be unlikely if APRV induced lung injury (Andrews et al., 2013a; Andrews et al., 2013b). There is a common mistake to assume linearity between macro-ventilatory parameters displayed on the ventilator and what’s happening in the micro-environment of the lung) where APRV actually produces minimal dynamic strain (Kollisch-Singule et al., 2018).

## Myth #9 –It is not Possible to Measure Driving Pressure in APRV

In a study by Zhou, et al. (2017), the authors stated, “In order to make the ∆P of APRV and LTV comparable, the ∆P of APRV was measured under the same conditions as with LTV” (*sic*). The authors temporarily changed modes in the APRV group to VAC to measure ∆P. The belief it is not possible to measure ∆P in APRV may be in part a result of a study by Kacmarek et al. (1995) using a single compartment test lung model comparing set vs auto-PEEP is often referenced and data extrapolated to imply APRV would produce heterogenous distribution of end expiratory pressure and EELV thereby rendering ∆P inaccurate. Unfortunately, this study does not provide details as to the duration used for expiratory pressure equilibration and more importantly a test lung does not capture the behavior of tissue stress recovery within the lung and chest wall tissues due to their viscoelastic behavior (Bates et al., 1988; Kochi et al., 1988). Subsequently, authors have extrapolated the test lung model data further by theorizing APRV would produce such heterogenous ventilation to increase risk of volutrauma and atelectrauma (Chatburn et al., 2016). However, biologic data exist that make these opinions inaccurate speculations.

In an animal model, IRV producing V_T_ 8–12 ml/kg was used to produce intrinsic PEEP (PEEPi) and compared static (PEEPi stat) vs dynamic (PEEPi Dyn) measurement (Hernandez et al., 1994). The PEEPi stat was measured using an expiratory hold for 5 s and PEEPi Dyn was measured using expiration to no flow state. Results show that PEEPi Dyn underestimates PEEPi stat only in acute non-homogeneous airway obstruction induced with methacholine (MCh) whereas PEEPi stat approximates PEEPi Dyn without MCh induced bronchospasm. This suggests airflow limitations, such as with bronchoconstriction, have the greatest effect on PEEPi Dyn [not PEEPi stat] measurements and the ability to estimate alveolar pressure and measure ∆P. Kollisch-Singule et al. (2016a), showed in a lung injury model with APRV that alveolar heterogeneity after lung injury was normalized to match normal uninjured alveolar size distribution thereby restoring alveolar homogeneity whereas LV_T_ was unable to normalize alveolar heterogeneity using external PEEP ranging from 5 to 24 cmH_2_O. In summary, APRV was superior to LV_T_ with external PEEP in producing uniform alveolar size and distribution after lung injury. In another study investigating alveolar to duct gas distribution ratio of conducting airway air space occupancy to alveolar air space occupancy in the distal airspace, APRV with T_Low_ set to *E*_FT_/*E*_FP_ 75% restored alveolar to duct gas ratios closely resembling pre-injury ratios. In contrast, LV_T_ and PEEP demonstrated inability to restore alveolar gas volume but progressively overdistended alveolar duct volume (Kollisch-Singule et al., 2014b). Lastly, in a sophisticated porcine lung injury model producing a “baby lung” with injured dependent and normal non-dependent lung regions, using APRV overdistension was simulated with P_High_ of 40 cmH_2_O. With APRV *E*_FT_/*E*_FP_ 75% neither atelectrauma nor overdistension resulted and produced the least injury to both normal and injured lung regions (Jain et al., 2017).

It has also been implied that APRV caused RV failure likely due to high ΔP from APRV with no supporting data (Chen et al., 2017). Conversely, ΔP was measured in 200 patients with a passive respiratory system such as post-operative patients (i.e., anesthesia or neuromuscular blocking agents) using a 4 s expiratory hold for equilibration (Andrews et al., 2019). Data was obtained and divided between four ventilator modes: 1) n = 86 VAC); 2) *n* = 28 PCV; 3) *n* = 74 APRV-TCAV™ as standard of care (S-APRV); and 4) *n* = 12 APRV-TCAV™ as a rescue mode (R-APRV) used when patients failed conventional ventilator modes. The ΔP was lowest in the S-APRV group compared to both the VAC group (p- value = 0.0010) and PCV group (*p*-value = 0.0002) but not statistically different than R-APRV group (*p*-value = 0.3379). Lastly, although Neumann et al., is often referenced regarding large pleural pressure swings with APRV increasing the risk of volutrauma and atelectrauma, they showed ∆P of mechanical breaths without spontaneous breathing decreased when T_Low_ was reduced from 2.5 to 0.5 s, such as that with the TCAV method where the *E*_FT_/*E*_FP_ is set to 75% (Neumann et al., 2002).

The power of mechanical ventilation, defined as the amount of energy transmitted from the ventilator to the lungs, is comprised of the driving pressure (delta P), V_T_ (delta V), RR, PEEP, and inspiratory gas flow such that a fast inspiratory delivery with a slow expiratory flow adds to the power (Gattinoni et al., 2016). The two variables found to be most associated with increased mortality are the ΔP and RR (Costa et al., 2021). Given the time settings used in APRV (T_High_ and T_Low_) ventilation is usually delivered with lower RR and ∆P. The T_Low_ and P_High_ depend on C_RS_, recruitability and because T_Low_ is brief, T_High_ is generally 1–1.5 x greater duration than conventional ventilation for a give RR. The extended T_High_ gradually restores lung volume improving gas exchange efficacy leading to progressive lower RR and when coupled with pairing V_T_ to C_RS_ (ie delta P), these time settings reduce key mechanical power variables. Mechanical power was found to be significantly reduced in APRV (11.9 J/min) compared to LV_T_ (20.7 J/min) in an experimental model of blast lung injury (Scott et al., 2020).

## Myth #10—Patients Must be Spontaneously Breathing for APRV to be effective/Spontaneous Breathing Durung APRV is Dangerous

This myth comes full circle with both pro and con statements about spontaneous breathing with APRV. It has been claimed that without spontaneous breathing, APRV is not effective, and the key physiologic advantages are lost (Dries and Marini, 2009; Modrykamien et al., 2011), yet others claim APRV can have a significant effect on work of breathing (WOB) and potential for harm with the cost of spontaneous breathing during APRV being markedly elevated. (Kallet, 2011; Daoud et al., 2012; Mireles Cabodevila and Kacmarek, 2016).

First, we address the claim regarding the benefits of APRV being lost without spontaneous breathing. Although spontaneous breathing may be facilitated with APRV, it is certainly not a prerequisite. Experimental and clinical data show benefits of APRV without spontaneous breathing (Roy S. et al., 2013; Roy SK. et al., 2013; Emr et al., 2013; Kollisch-Singule et al., 2014a; Kollisch-Singule et al., 2014b; Kollisch-Singule et al., 2016a; Kollisch-Singule et al., 2016b; Jain et al., 2017). Because APRV with the TCAV™ method can prevent or halt the progression of VILI and subsequently stabilize the lung, it can be used successfully even in brain dead donors who are obviously not spontaneously breathing (Roy et al., 2017; Koch et al., 2019). In fact, in a study of donors managed with APRV, lung utilization occurred more in the APRV group (84%) than the VAC group (18%) (*p* < 0.001) (Hanna et al., 2011). Additionally, organ recovery data demonstrate an increase in overall organs recovered in donors where APRV has been used during donor management (Roy et al., 2017; One Legacy. 2022).

Second, claims that spontaneous breathing during APRV is harmful and associated with an increased WOB have no supporting data nor validation of this occurring with greater frequency in APRV than with any other ventilator mode (Kallet 2011; Daoud et al., 2012; Mireles Cabodevila and Kacmarek, 2016; Yoshida et al., 2017). The 2002 Neumann, et al. paper (Neumann et al., 2002) is cyclically referenced to indict spontaneous breathing with APRV as always being harmful. Misquoted as a clinical trial of 35 patients (Myers and Macintyre, 2007), this is in fact is an observational study with a mixed population of 28 patients: COPD (25%), Acute Lung Injury (32%) (moderate ARDS by Berlin criteria), and Non-specific Pathology (43%). In the Neumann protocol, the Dräger Evita 1 ventilator was used in the BIPAP mode, which has a trigger and flow termination fixed to 25% and could increase WOB for the COPD patients. The P_High_ was stepwise increased until the patient stopped breathing spontaneously and then reduced by 25% of an unknown P_High_ to induce spontaneous breathing, potentially causing derecruitment and distress. In fact, the authors express concern in the manuscript that releasing the pressure from P_High_ to P_Low_ [even for a brief duration] could provoke lung collapse. However, they proceed to use a protocol that precipitously reduced P_High_ and extended the duration at P_Low_ at the onset of data collection, potentially creating a greater magnitude of collapse and respiratory distress. The authors also point out that: “Thus, if such very short release times are used in critically ill patients, adequate ventilatory support has to be assured”. Additionally, they state: “This can either be obtained by an increase of P_High_ to increase the driving pressure of the mechanical breaths or an increase of the release cycles” (i. e., forcing patients to breathe spontaneously at the onset of distress would most likely uncover excessive WOB). Lastly, because each ventilatory setting was used for only ∼30 min, the authors make the acknowledgement: “Thus, no conclusion can be drawn about potential long-term effects (e.g., development of respiratory muscle fatigue or improvement of oxygenation) of the respiratory settings used in the present study.”

This paper is also hindered by numerous methodologic and statistical issues such as a small sample size with lack of power and high standard deviations, particularly with the transpulmonary data [used to assess WOB] and Simpson’s paradox conflating a spectrum of lung pathologies. For instance, it is unlikely that optimal ventilator parameters for patients with ALI would be identical to that of patients with COPD. No major adverse effects were observed with brief T_Low_ settings on hemodynamics, the ability to spontaneously breath or gas exchange in any of the three patient groups. Most importantly the authors never conclude that APRV increased WOB or is harmful and the actual conclusion of the study was: “Airway pressure release ventilation is an open system which allows patients to maintain the “time control” over the respiratory cycle independent of the chosen duration for P_High_ and P_Low_.”

Excessive WOB is important, yet often underappreciated, in patients during mechanical ventilation (Yoshida et al., 2017). Unfortunately, dyspnea and distress are common and occur even in conventional modes of mechanical ventilation (Schmidt et al., 2011, 2014). Dyspnea during mechanical ventilation is often associated with anxiety, pain as well as inappropriate ventilator settings (Schmidt et al., 2011, 2014; Worsham et al., 2021) and has been linked with higher mortality during a patient’s hospital admission in addition to the 2 years after discharge (Stevens et al., 2021). Further, dyspnea after extubation has been associated with an increased risk of recurrent respiratory failure (Dres et al., 2021). Patients can also experience mental discomfort and a form of post traumatic distress syndrome (PTSD) during mechanical ventilation (Schmidt et al., 2014; Worsham et al., 2021; Schwartzstein and Campbell, 2022). However, despite the risk of spontaneous breathing causing dyspnea and excessive breathing efforts, absence of spontaneous breathing during mechanical ventilation is equally detrimental. In fact, both excessive breathing or elimination of spontaneous breathing prolong duration of mechanical ventilation and impact patient outcome (Goligher et al., 2018). When considering spontaneous breathing it is important to understand it should never be viewed as a binary event–i.e., only described as present or absent - or depend on the solely on the acronym of the ventilator mode - but more importantly depend on the ventilator interactions with the patient’s respiratory system.

Autonomic spontaneous breathing originates and is controlled in the brainstem. The brainstem controls the drive to breath and the respiratory muscles’ output, which are regulated by the abundant feedback of chemical and mechanoreceptors that must be satiated in order to attain the many benefits and efficiency of spontaneous breathing. Hierarchical control of factors such as gas exchange, lung volume, C_RS_, degree of respiratory muscle dysfunction, and diaphragm position all affect the patient’s ability to perform acceptable spontaneous breathing without excessive WOB. No one mode of mechanical ventilation including APRV, or ventilatory condition for that matter, can guarantee the promise of not inducing excessive WOB. Rather it is the clinician’s role to support, select and prepare the patient who is capable of undergoing unharmful spontaneous breathing and eliminate complications such as VILI and ventilator induced diaphragm dysfunction (Goligher et al., 2015; Goligher et al., 2018; Goligher et al., 2020). Since both excessive and absence of spontaneous breathing are now recognized as a detriment to patient outcome, more than ever this demands that optimal approaches and understanding of the respiratory system is required. We can no longer be complacent allowing patients to stagnate on the ventilator while admiring ventilatory parameters, pulse oximetry and arterial blood gas.

Increased inspiratory effort may have deleterious effects on the lung—this is the concept of patient-self-inflicted lung injury (P-SILI) (Brochard et al., 2017). Since P-SILI is determined by the changes in transpulmonary pressure which are not dependent on a specific ventilatory mode and can occur even in the absence of a ventilator. Therefore, appropriately set APRV and patient selection should not expose patients to a higher risk of P-SILI. Because high flow demands, and air hunger are the worst form of dyspnea it is important to understand which ventilator settings may relieve or worsen distress. Air hunger is reduced with increased levels of PEEP by increasing EELV (Vovk and Binks, 2007) For example, spontaneous breathing at low levels of PEEP is associated with greater lung and diaphragm injury whereas spontaneous breathing at higher levels of PEEP is protective (Yoshida et al., 2016; Morias et al., 2018). Physiologically, as lung volume increases mechanical receptors provide feedback to the brainstem, which depresses inspiratory effort and signals expiratory muscles for active exhalation if needed (Road and Leevers, 1988; Road and Leevers, 1990; Torres et al., 1993). Conversely, at low lung volume expiration is suppressed with maximum inspiratory drive activated (Dempsey 1994). Lung volume and diaphragm contraction are optimally positioned for breath initiation at FRC. Lung volume changes from FRC alter the curve of the diaphragm and change the force generating capabilities (Road and Leevers, 1988; Road and Leevers, 1990). As lung volume increases, the diaphragm curvature diminishes (flattens) such that the force generating capacity decreases (ie force-length relationship) (Braun et al., 1982; Road and Leevers, 1988; Road and Leevers, 1990). The opposite is true at low lung volume where both the force generation of the diaphragm is maximal and is synergized with the high inspiratory drive at the brainstem (Yoshida et al., 2017). In fact, a case report by Kallet et al. (1999) illustrates this in a patient transitioning from PCV to VAC LV_T_ for the ARMA trial who developed rapid onset of negative pressure pulmonary edema and decrease in C_RS_ and subsequent rapid resolution of the pulmonary edema with the removal of LV_T_. This led the authors to say: “……exacerbation of acute pulmonary edema coincided with the institution of a lung-protective strategy. The fact that pulmonary edema quickly appeared and resolved with the institution and removal of low V_T_ ventilation strategy led us to suspect that vigorous inspiratory efforts were responsible for the sudden deterioration in the patient’s cardiorespiratory status.” What these authors document is well understood in the physiology of dyspnea and the brainstem’s control over lung volume. Many respiratory pathologies such as impaired gas exchange, lung injury and activation of chest wall and other receptors can increase respiratory drive and the sensation of dyspnea, reflexively stimulating further attempts to increase V_T_. Patients with ARDS receiving LV_T_ strategy are more likely to experience air hunger, dyspnea and remain on the ventilator for a prolonged period of time potentially increasing the risk for PTSD symptoms (Schmidt et al., 2011, 2014; Worsham et al., 2021; Schwartzstein and Campbell, 2022). However, the LV_T_ strategy opposes the inherent physiologic mechanism to resolve dyspnea–increased lung volume (Worsham et al., 2021). Additionally, lower lung volumes position the diaphragm to generate high force and pressure to satisfy the high inspiratory drive. Alternatively, spontaneous breathing in APRV with the TCAV™ method targets breath initiation at or slightly above FRC. Once FRC is reestablished, the CPAP phase of APRV decreases inspiratory effort and air hunger provided proper lung volume and flow demands are met (Gregory et al., 1971; Gherini et al., 1979). Inspiratory efforts are usually minimal as the typical breathing pattern is to defend lung volume where expiratory muscles provide inspiratory assistance (Torres et al., 1993). The CPAP Phase (or P_High_) permits lung volume titration that allows the patient to traverse between active exhalation and minimal inspiratory effort. Coupled with the open breathing system, the ability of the patient to have unrestricted spontaneous breathing preserves the neural inspiratory time making the patient less distressed. These features improve patient-ventilator interaction during properly set APRV and properly selected patients to satiate respiratory demand, allow control over ventilation and increase comfort suggesting that APRV may facilitate spontaneous breathing with an appropriate level of work.

## Misconception of APRV Trials

We believe we have provided sufficient evidence to demystify 10 myths that are perpetuated in the literature through unsubstantiated statements, but other myths remain that were not addressed in this review. We do, however, wish to discuss the frequent objection with the use of APRV, which is the recall bias of the negative studies. Although there is no multi-center RCT to date showing APRV is superior to LV_T_, there is equally no multi-center RCT to date showing LV_T_ to be superior to APRV. In fact, recent meta-analyses suggest a point estimate in favor of APRV–although the heterogeneity is high (Lim and Litton, 2019; Zhong et al., 2020).

Three trials are often highlighted as APRV failures. It is important to note that prior to the 2000 ARDSNet ARMA trial (ARDSNet 2000), three LV_T_ studies using various methods failed to show any benefit (Brochard et al., 1998; Stewart et al., 1998; Brower et al., 1999). Subsequently, it took the 41 million-dollar ARMA trial comparing two methods of setting V_T_ in VAC with the LV_T_ method (6 ml/kg) vs (HV_T_) method (12 ml/kg) (ARDSNet 2000) to show a reduction in mortality. When reading beyond the ‘headlines’ in the alleged negative three trials with APRV, a critical review reveals the following:

1) The Hirshberg et al. (2018) trial was stopped for futility but not because the mode APRV was futile or for patient harm. The goal of the study was to conform APRV V_T_ to ≤6 ml/kg similar to that of LV_T_ and was stopped because this goal could not be met. There were three groups: 1) LV_T_ with VAC targeting V_T_ 6 ml/kg (n = 17); 2) APRV targeting V_T_ 6 ml/kg (n = 18); 3) APRV with no target V_T_ (n = 17). Allowing the T_Low_ to be adjusted to 50–75% EF_T_/EF_P_ could explain the V_T_ exceeding 12 ml/kg. Interestingly, even with V_T_ exceeding 12 ml/kg in both APRV groups, there was no increase in barotrauma or mortality. Besides the V_T_ goal not being met, there was no significant difference in hemodynamics or vasoactive requirements, barotrauma rates, sedation or NMBA use, reintubation, ventilator-free days, hospital mortality or ICU length of stay.

2) The Ibarra-Estrada, et al. (2022) trial has been referred to as the APRV study of CARDS patients that was stopped for mortality. Here are the facts. After four episodes of barotrauma, a review by the data safety monitoring board recommended stopping recruitment for patients with COVID-19, although the decision was not unanimous. Prior to the study being stopped, there was no difference in mortality or difference in barotrauma rates between the groups, which was the impetus to recommend stopping the trial. Shrestha et al. (2022) showed in a systematic review and meta-analysis that increased barotrauma incidence was associated with increasing disease severity in COVID-19 and not linked with a particular mode. We previously reviewed the Ibarra-Estrada, et al. (2022) study and the incidence of hypercapnia in the APRV group in detail where the RR was lower than in the LV_T_ group. The authors admit clinicians were reluctant to use a T_High_ lower than the typical 4–6 s that is used in less ill patients, thereby decreasing the set RR in the APRV group.

3) The Ganesan et al., trial reports APRV was associated with a trend toward higher mortality compared to LV_T_ when used as a primary ventilation strategy in children with ARDS (Ganesan et al., 2018). However, patients in the APRV group with a primary cause of lung injury/ARDS had a longer duration of respiratory complaints and more cases of severe ARDS at enrollment indicating a sicker group of patients. Further there was an increased number of contaminated cases in the APRV group, which was defined as those who required an alternative mode of ventilation but is not fully explained. Conversely, the trend of barotrauma rates, use of sedation and analgesia and hemodynamic instability was lower in the APRV group. The authors point out that spontaneous breathing is a prerequisite for APRV, which we previously reviewed is not necessary. However, similar to the Ibarra-Estrada et al. (2022) study the T_High_ was set at 4.0 s, which would significantly decrease the set RR and force patients to assume the majority of the total MVe. A T_High_ of 4.0 s is exceedingly high in pediatric ARDS patients when this would translate to a RR of [the highest] 14 b/min as the T_Low_ on the ventilator used (Hamilton Galileo) cannot be set <0.2 s. Not only will a T_High_ of 4.0 s create a prolonged CPAP Phase where the patient assumes a greater portion of the total MVe and metabolic load, but without setting the T_Low_ to achieve E_FT_/E_FP_ of 75%, alveolar instability and RACE is never stopped so that recruitment can begin. Lastly, if a reliable Pplat was not attained in this study, the P_High_ was initially set at 15cmH_2_O and adjusted incrementally up to a P_High_ of 28 cmH_2_O to achieve correlate P/F ratios. However, if the V_T_ exceeded 6–7 ml/kg ideal body weight (IBW), the P_High_ was decreased, which may have led to further derecruitment, loss of surface area and subsequently worsening hypercarbia and excessive WOB. In summary, alveolar instability may never have been halted leading to the worse P:F ratios and subsequent trend to increased mortality in the APRV group could have resulted from settings where the T_High_ was most likely much too high for a pediatric ARDS patient, the T_Low_ was possibly not set to achieve 75% if <0.2 s was required and the P_High_ reduced if the V_T_ exceeded 6–7 ml/kg IBW. These and additional statistical analysis issues have been addressed by other authors (Daxon 2018).

## Summary

Science should be based on evidence. Negative and sometimes Pavlovian responses regarding APRV are published without supporting data. Some authors even declare “APRV is a dangerous mode” (Kallet et al., Respir. Care, 2011, 56(2), 190–203), “there is no reason to consider this approach to ventilator support” and “APRV is the devil’s spawn” (Mireles Cabodevila and Kacmarek, 2016) without any science to validate these claims. In fact, some of the most enthusiastic objections towards APRV were followed by the admission of little to no clinical experience using the mode (Mireles Cabodevila and Kacmarek, 2016). It would be “anti-science” to ignore or condemn new data because they do not fit one’s prior ideas. The appropriate approach would be to review and consider all scientific and clinical information carefully to understand it wholly and become a useful critic.

The goal of this review was to highlight the most published myths and misconceptions and evaluate if any of these claims are supported scientifically. What we found were recurring statements that lack support and that many were recycled logical fallacies. Although APRV is far from being adequately studied scientifically, the TCAV™ method highlights non-traditional concepts of lung management that warrant further exploration to expand our knowledge of the lung in general and lung—ventilator interactions. We believe we have shown APRV is not an overly complex mode that is too difficult to understand, is distinguishable from IR-PCV, does not itself create barotrauma or volutrauma and itself generate high V_T_, does not cause increased RV strain or unsafe auto-PEEP, does not cause alveolar collapse if P_Low_ is set to 0 cmH_2_O, can control PaCO_2_ and set a sufficient RR, can obtain a ΔP and can be used whether the patient is or is not spontaneously breathing. Science has always benefited from competitive ideas, debate, and the constant refinement of our concept all of which are advanced by knowledgeable critics. Unfortunately, misinformation is the nemesis of science.

As Albert Einstein said, “The important thing is to never stop questioning”.
